# SIRT1 regulates cardiomyocyte alignment during maturation

**DOI:** 10.1242/jcs.259076

**Published:** 2022-04-01

**Authors:** Yi Fang, Wei Fan, Xiaojiang Xu, Agnes K. Janoshazi, David C. Fargo, Xiaoling Li

**Affiliations:** ^1^ Signal Transduction Laboratory, National Institute of Environmental Health Sciences, Research Triangle Park, NC 27709, USA; ^2^ Integrative Bioinformatics, National Institute of Environmental Health Sciences, Research Triangle Park, NC 27709, USA; ^3^ Fluorescence Microscopy and Imaging Center, National Institute of Environmental Health Sciences, Research Triangle Park, NC 27709, USA

**Keywords:** SIRT1, Cardiomyocyte maturation, Alignment, Contraction, Chemotaxis

## Abstract

Cardiomyocyte elongation and alignment, a critical step in cardiomyocyte maturation starting from the perinatal stage, is crucial for formation of the highly organized intra- and inter-cellular structures for spatially and temporally ordered contraction in adult cardiomyocytes. However, the mechanism(s) underlying the control of cardiomyocyte alignment remains elusive. Here, we report that SIRT1, the most conserved NAD^+^-dependent protein deacetylase highly expressed in perinatal heart, plays an important role in regulating cardiomyocyte remodeling during development. We observed that SIRT1 deficiency impairs the alignment of cardiomyocytes/myofibrils and disrupts normal beating patterns at late developmental stages in an *in vitro* differentiation system from human embryonic stem cells. Consistently, deletion of SIRT1 at a late developmental stage in mouse embryos induced the irregular distribution of cardiomyocytes and misalignment of myofibrils, and reduced the heart size. Mechanistically, the expression of several genes involved in chemotaxis, including those in the CXCL12/CXCR4 and CCL2/CCR2/CCR4 pathways, was dramatically blunted during maturation of SIRT1-deficient cardiomyocytes. Pharmacological inhibition of CCL2 signaling suppressed cardiomyocyte alignment. Our study identifies a regulatory factor that modulates cardiomyocyte alignment at the inter-cellular level during maturation.

## Teaser

**Summary:** Analyses of mouse embryos and cardiomyocytes differentiated from human embryonic stem cells show that SIRT1 regulates cardiomyocyte alignment during development, in part through chemotaxis.

## INTRODUCTION

As a blood-pumping organ, the heart is extremely well organized in its structure to achieve the best contractile efficiency. The adult cardiac myocytes are elongated and aligned along a common axis, which is critical for rapid electrical propagation and the uniaxial alignment of sarcomeres, both of which contribute to spatially and temporally ordered contraction (McCain and Parker, 2011). Arising from cardiac progenitors, embryonic cardiomyocytes are round and polygonal in shape, and robust in proliferation capacity, and their myofibrils are not oriented in the cytoplasm (Hirschy et al., 2006). Embryonic cardiomyocytes undergo a process termed maturation, which starts at embryonic stages and continues until adulthood, to become adult cardiomyocytes. The maturation process includes elongation and alignment of cardiomyocytes and their myofibrils, polarization of intercalated discs to connect cardiomyocytes to neighboring cardiomyocytes, and invagination of transverse tubules into the cells during postnatal development (Di Maio et al., 2007; Hirschy et al., 2006; Ziman et al., 2010). Major advances have been made in understanding how cardiomyocytes are differentiated from stem cells over the past decades, yet the mechanisms that regulate the maturation process remain unclear.

Silent mating type information regulation 2 homolog 1 (SIRT1) is a NAD^+^-dependent protein deacetylase (Class III) that plays critical roles in multiple cellular processes, including metabolism, inflammation, stress response and stem cell functions (Han et al., 2008; Houtkooper et al., 2012; Tang et al., 2014). SIRT1 is also important for animal development. Systemic deletion of SIRT1 in mice leads to severe developmental defects in multiple tissues, including intrauterine growth retardation, developmental defects of the retina and heart, defective germ cell differentiation and neonatal lethality (Cheng et al., 2003; McBurney et al., 2003; Wang et al., 2008). Particularly, SIRT1 is highly expressed in perinatal/neonatal hearts compared to fetal and adult hearts (Planavila et al., 2012). SIRT1-null embryos display septal defects in heart, and surviving adults on the mixed genetic backgrounds develop dilated cardiomyopathy with smaller cardiomyocytes and mitochondrial dysfunction (Cheng et al., 2003; Planavila et al., 2012). Despite these studies, however, how SIRT1 regulates heart development and function is still largely unknown.

## RESULTS

### SIRT1 deficiency disrupts alignment of perinatal cardiomyocytes and cardiac myofibrils *in vivo*

**Fig. 1.**
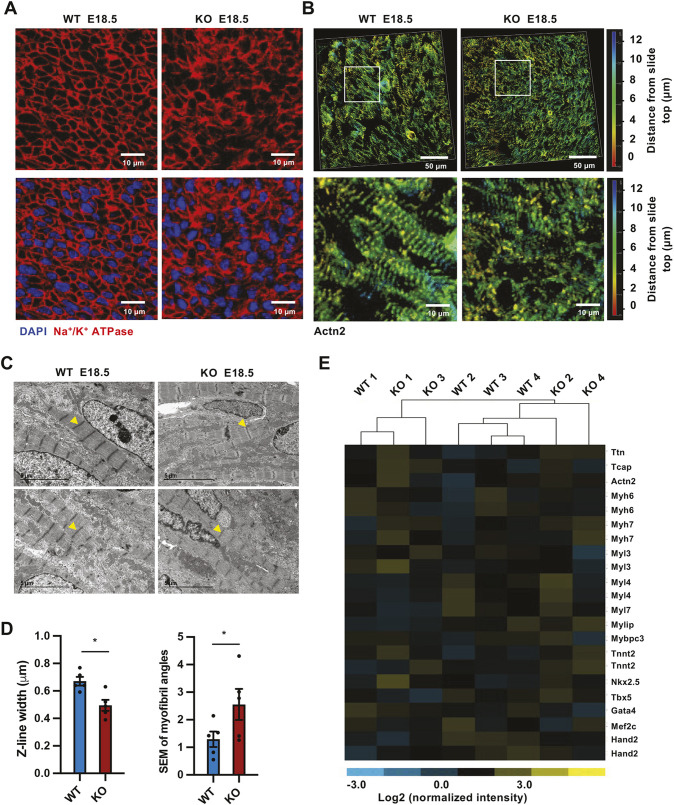
**SIRT1 deficiency disrupts cardiomyocyte and myofibril alignment *in vivo*.** (A) E18.5 SIRT1 whole-body germ-line knockout (SIRT1 KO) mouse hearts have misaligned cardiomyocytes. Cross sections towards the base of the left ventricle of hearts from wild-type (WT) and SIRT1 KO embryos at E18.5 were stained with anti-Na^+^/K^+^ ATPase antibodies (*n*=4 embryos/genotype, representative images are shown). (B) WT and SIRT1 KO mouse hearts display distinct myofibril structures. Cross sections towards the base of the left ventricle of hearts from WT and SIRT1 KO embryos at E18.5 were stained with anti-ACTN2 antibodies and analyzed by 3D imaging (*n*=4 embryos/genotype, representative images are shown). (C,D), SIRT1 KO mouse hearts have reduced Z-line width and impaired alignment of myofibrils. Hearts from E18.5 WT and SIRT1 KO embryos were processed for electron microscopy analysis of sarcomere structures. The width of the Z-line and the angle of individual myofibrils were measured and compared (*n*=5 individual embryos with total of 35 observation fields/genotype, representative images from two pairs of embryos are shown in C, *P<0.05, Mann-Whitney test, values represent mean±s.e.m.). Arrowheads in Fig. 1C indicate Z-lines. (E) The mRNA levels of genes critical for cardiomyocyte differentiation and contraction were not significantly different between WT and SIRT1 KO E18.5 hearts. The total RNAs of WT and SIRT1 KO E18.5 hearts were analyzed by mouse whole-genome microarrays (*n*=4 embryos/genotype).

To investigate the roles of SIRT1 in regulating heart development and function, we first analyzed the heart development of wild-type (WT) and SIRT1 whole-body germ-line knockout (SIRT1 KO) mice on the C57BL/6J background. SIRT1 KO mouse embryonic hearts had no clear gross anatomical anomalies compared to WT hearts before embryonic day (E)14.5 on this genetic background (data not shown). However, SIRT1 KO hearts exhibited irregular distribution of their cardiomyocytes compared to WT hearts when analyzed on E18.5, as revealed by immunofluorescent staining of a plasma membrane protein, Na^+^/K^+^ ATPase (Fig. 1A). E18.5 SIRT1 KO hearts also displayed abnormal morphology of sarcomeres (Fig. 1B–D), the fundamental contractile unit of striated muscle tissue including cardiac muscle. A sarcomere is delimited by Z-lines, in which a key sarcomeric protein, α-actinin 2 (ACTN2), cross-links actin with a giant molecular spring, titin (TTN), and rejoins adjacent sarcomeres (Fig. S1A). Immunofluorescent staining of ACTN2 unveiled that myofibrils, the elongated contractile threads consisting of numerous sarcomeres, were short and not well organized in the hearts from E18.5 SIRT1 KO mouse embryos compared to those from WT embryos in three-dimensional (3D) imaging analysis, in which a color scale was used to indicate the cell depth (Fig. 1B). Electron microscopy analyses of the sarcomere ultrastructure further showed that the Z-lines in SIRT1 KO E18.5 hearts were significantly shorter, and the muscle fibers in these hearts were also significantly less organized, than those in WT hearts (Fig. 1C,D; Fig. S1B). These observations indicate that SIRT1 deficiency impairs the alignment of cardiomyocytes and their myofibrils, a critical maturation step of cardiomyocytes in perinatal hearts.

SIRT1 is a predominately nuclear NAD^+^-dependent protein deacetylase in most cell types, in which it controls chromatin structures and gene expression through metabolic and epigenetic regulation of post-translational modification of histones and transcription factors (Houtkooper et al., 2012; Imai and Guarente, 2010; Tang et al., 2017). To dissect the possible molecular mechanisms underlying SIRT1 deficiency-induced misalignment of cardiomyocytes and myofibrils, we determined the transcriptomes of E18.5 WT and SIRT1 KO hearts by microarray. Surprisingly, the expression levels of genes involved in cardiomyocyte differentiation and function were not significantly changed in E18.5 SIRT1 KO hearts compared to WT hearts (Fig. 1E; Table S1), and pathways that mediate cardiac development and differentiation were also not enriched in the top significantly altered gene list between SIRT1 KO and WT mouse hearts when analyzed by Ingenuity Pathway Analysis (IPA) (Table S2). These findings suggest that SIRT1 does not regulate cardiomyocyte/myofibril alignment through transcriptional regulation of sarcomeric/cardiogenic genes.

### SIRT1 deficiency impairs cardiomyocyte alignment and beating *in vitro*

**Fig. 2.**
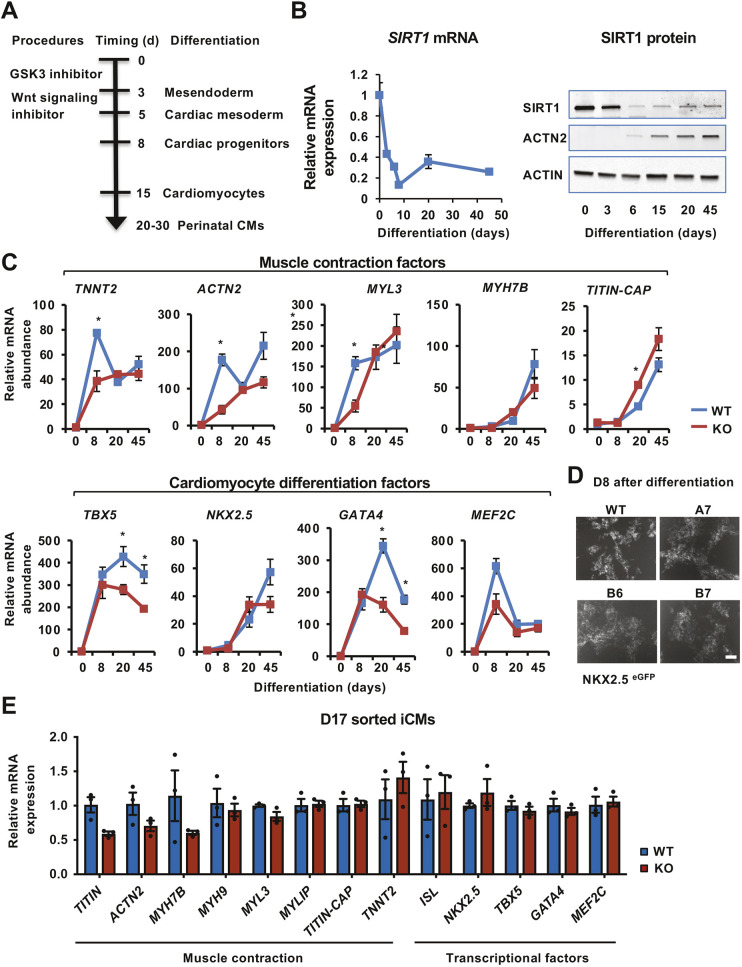
**SIRT1 deficiency does not impair differentiation of cardiac progenitors from human embryonic stem cells (hESCs).** (A) A strategy to induce cardiomyocytes from hESCs. (B) The relative mRNA and protein levels of SIRT1 were analyzed at indicated times after differentiation by quantitative PCR (qPCR) with *GAPDH* as a loading control (left, *n*=3 biological replicates, values represent mean±s.e.m.) and an anti-SIRT1 antibody (right). (C) The induction of key cardiomyocyte differentiation factors and muscle contraction factors in WT and SIRT1 KO cardiomyocytes. WT and SIRT1 KO mel1 hESCs were induced for cardiomyocyte differentiation, and total RNA was collected on Day (D)0, D8, D20 and D45, and processed for qPCR analysis of the indicated cardiogenesis genes (*n*=3 independent lines/genotype, *P<0.05, Student's *t*-test, values represent mean±s.e.m.). (D) Induction of NKX2.5^eGFP^-positive cardiac progenitors is not blocked in SIRT1 KO cells. WT and SIRT1 KO mel1 hESCs were subjected to cardiac differentiation, and the NKX2.5^eGFP^ signals were analyzed at D8 after the differentiation. Scale bar: 200 µm. (E) Isolated WT and SIRT1 KO induced cardiomyocytes (iCMs) display no obvious differences in the expression levels of key cardiogenesis genes at D17 after differentiation. D17 GFP-positive iCMs differentiated from WT and SIRT1 KO mel1 cells were sorted by fluorescence-activated cell sorting, and the expression of indicated genes was analyzed by qPCR with *GAPDH* as a loading control (*n*=3 independent lines, values represent mean±s.e.m.).

To better understand how SIRT1 modulates cardiomyocyte alignment and maturation at the cellular and molecular levels, we took advantage of an *in vitro* differentiation system in which cardiomyocytes can be efficiently differentiated from human embryonic stem cells (hESCs) (Fig. 2A) (Lian et al., 2012, 2013). By recapitulating cardiomyocyte differentiation and remodeling *in vivo*, this system confers an alternative way to study human heart development, which is otherwise unlikely. It also provides a reliable source of cardiomyocytes for studying cardiomyocyte morphology and function because primary cardiomyocytes are not proliferative *in vitro*.

We first analyzed the expression levels of *SIRT1* and a number of genes critical for cardiogenesis during *in vitro* cardiac differentiation of the *NKX2.5^eGFP/W^* mel1 hESC line (Fig. 2B,C), in which the expression of eGFP is under control of the endogenous *NKX2.5* (also known as *NKX2-5*) locus (Elliott et al., 2011). NKX2.5 is a homeobox-containing transcription factor that is expressed in early cardiac mesoderm cells to regulate heart development from the cardiac crescent in humans. The NKX2.5^eGFP/W^ in mel1 cells therefore can serve as a reporter to track cardiac progenitors and cardiomyocytes following induction of differentiation (Elliott et al., 2011). As shown in Fig. 2B, the mRNA and protein levels of SIRT1 were dramatically reduced during the differentiation from hESCs to cardiac progenitors [Day (D)0-D8], which is consistent with our previous observations that SIRT1 is important for the maintenance of pluripotent embryonic stem cells (Tang et al., 2014, 2017). In contrast, the expression of a number of genes critical for cardiogenesis or controlling cardiac muscle contraction were massively induced with different dynamics during *in vitro* cardiac differentiation of mel1 hESCs (Fig. 2C, WT). Intriguingly, *SIRT1* was markedly re-expressed at the perinatal cardiomyocyte stages after initial reduction at the progenitor stage (Fig. 2B, D20-D45), suggesting that SIRT1 may have important functions in perinatal cardiomyocytes.

To test the importance of SIRT1 in regulation of cardiomyocyte differentiation and functions, we utilized three previously generated independent mel1 hESC clones with the *SIRT1* gene deleted using the CRISPR/Cas9 gene-editing technology, A7, B6 and B7 (Fan et al., 2021). Deletion of SIRT1 in mel1 hESCs did not have significant impact on cell morphology and proliferation when cells were maintained in a hESC maintenance medium (not shown). When subjected to *in vitro* cardiomyocyte induction, SIRT1-null mel1 cells were as efficiently induced into NKX2.5^eGFP^-positive progenitors as the WT cells on D8 (Fig. 2D), indicating that deletion of SIRT1 in hESCs does not block their differentiation into cardiac progenitors. Moreover, despite reduced induction of several cardiomyocyte differentiation factors, such as TBX5 and GATA4, during the course of cardiomyocyte differentiation (Fig. 2C, KO), sorted NKX2.5^eGFP^-positive SIRT1 KO induced cardiomyocytes (iCMs) on D17 displayed no significant differences in the expression levels of many key genes mediating cardiomyocyte differentiation and contraction compared to WT iCMs except for a minor reduction in *TTN* (Fig. 2E), indicating that SIRT1 deficiency has only modest effects on the differentiation of cardiac progenitors into cardiomyocytes *in vitro*.

**Fig. 3.**
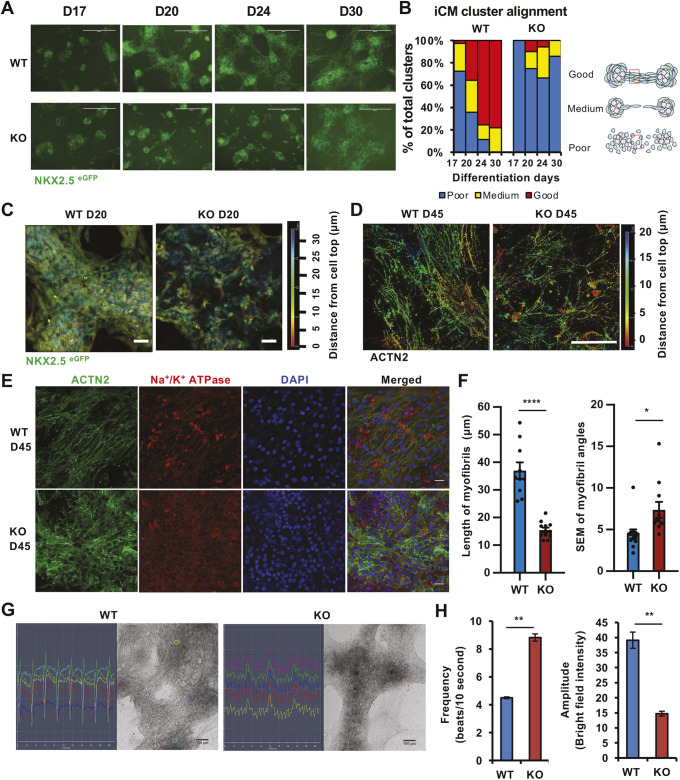
**SIRT1 deficiency impairs myofibril alignment and cardiomyocyte beating *in vitro*.** (A) WT and SIRT1 KO iCMs exhibit different morphology and alignment. WT and SIRT1 KO mel1 hESCs were induced for cardiomyocyte differentiation. The NKX2.5^eGFP^-positive iCM clusters were imaged on D17, D20, D24 and D30. Scale bars: 400 µm. (B) Quantification of morphology and alignment of WT and SIRT1 KO iCMs. The morphology of iCM clusters was categorized into three groups (as indicated by the cartoon on the right): elongated smooth fiber-like structures connecting most adjacent clusters and/or surrounding clusters (good); some cells connecting adjacent clusters or surrounding clusters (medium); few or no cells (fiber-like structures) connecting adjacent clusters or surrounding clusters (poor) (*n*=60–123 images from three biological replicates/genotype at each time point). (C) 3D images showing the different morphology and alignment of WT and SIRT1 KO iCMs on D20. Scale bars: 50 µm. (D) 3D images showing that WT and SIRT1 KO iCMs have distinct myofibril alignment. D45 WT and SIRT1 KO cardiomyocytes were stained with anti-ACTN2 antibody, and the *z*-stack 3D images were depicted with different colors for depth. Scale bar: 100 µm. (E) Cellular and myofibril alignment is disorganized in SIRT1 KO cardiomyocytes. D45 WT and SIRT1 KO cardiomyocytes were stained with anti-ACTN2 antibody and anti-Na^+^/K^+^ ATPase antibody. Red boxes in the cartoon in B indicate where cells were selected for myofibril analysis. Scale bars: 100 µm. (F) Quantification of WT and SIRT1 KO cardiomyocyte myofibril lengths and angles. The length and angle of individual myofibrils were measured, and the s.e.m. of the angles calculated and compared between WT and KO iCMs (*n*=10-12 images from three biological replicates/genotype, *P<0.05, ****P<0.0001, Student's *t*-test, values represent mean±s.e.m.). (G) WT and SIRT1 KO iCMs have different beating patterns. The beating patterns of five different locations are shown for each group. (H) Quantification of beating frequency and amplitude of WT and SIRT1 KO iCMs (*n*=50 WT and 90 KO analyzed areas from three independent cell lines/genotype, **P<0.01, Student's *t*-test, values represent mean±s.e.m.).

To further evaluate the possible involvement of SIRT1 in regulation of cardiomyocyte maturation and function at late stages, as suggested by our observations *in vivo* (Fig. 1), we monitored the morphology of WT and SIRT1 KO iCMs in culture dishes for 30–45 days after initiation of cardiomyocyte differentiation. As shown in Fig. 3A and B, both WT and SIRT1 KO iCMs emerged primarily as clumpy clusters before D20. By D20, WT iCMs appeared to migrate out of the clumps and extend either around the clumps or reach the nearby clusters. By D24, close to 80% of WT iCMs formed good alignment along a common direction, and this well-aligned fraction was maintained by D30, indicating that D17–D24 was a critical window for cardiomyocytes to remodel for alignment *in vitro* (Fig. 3A,B, WT). However, this directional growth and arrangement was largely absent in SIRT1 KO iCM clusters, which either failed to migrate or grew in random directions even after long-term culture (Fig. 3A,B, KO). When 3D images were taken with the cell depth decorated by a color scale, NKX2.5^eGFP^-positive WT iCMs appeared to be neatly and closely packed (Fig. 3C, WT D20). However, SIRT1 KO cells were sparsely and irregularly arranged (Fig. 3C, KO D20). Three-dimensional images of ACTN2 staining in D45 cells further showed that WT cardiomyocytes formed elongated myofibrils along one axis, whereas myofibrils in SIRT1 KO cells were randomly oriented (Fig. 3D). When further analyzed on D45 by immunofluorescent staining of ACTN2 and Na^+^/K^+^ ATPase, WT iCMs were aligned along the axis of myofibrils; however, similar to their short and disorganized myofibrils, SIRT1 KO iCMs were randomly distributed without clear direction (Fig. 3E,F). These morphological differences and the myofibril alignment phenotypes observed in iCMs were consistent with our observations *in vivo* (Fig. 1), confirming that SIRT1 deficiency impairs the elongation and uniaxial alignment of cardiomyocytes and their myofibrils *in vitro* and *in vivo*.

Further time-lapse video analyses revealed that the impaired structures in SIRT1-deficient iCMs are associated with marked disruptions of their beating patterns. SIRT1 KO iCMs beat much faster than WT iCMs, but with smaller amplitudes and more irregular rhythms (Fig. 3G,H). Therefore, in contrast to the homogeneous and robust beating of the WT cells, the beating of SIRT1 KO cells was like a fibrillation. Collectively, our findings demonstrate that SIRT1 is crucial in regulating the elongation and alignment of cardiomyocytes and myofibrils, as well as the cardiomyocyte contractility and beating patterns.

### SIRT1 regulates cardiomyocyte remodeling at late developmental stages

**Fig. 4.**
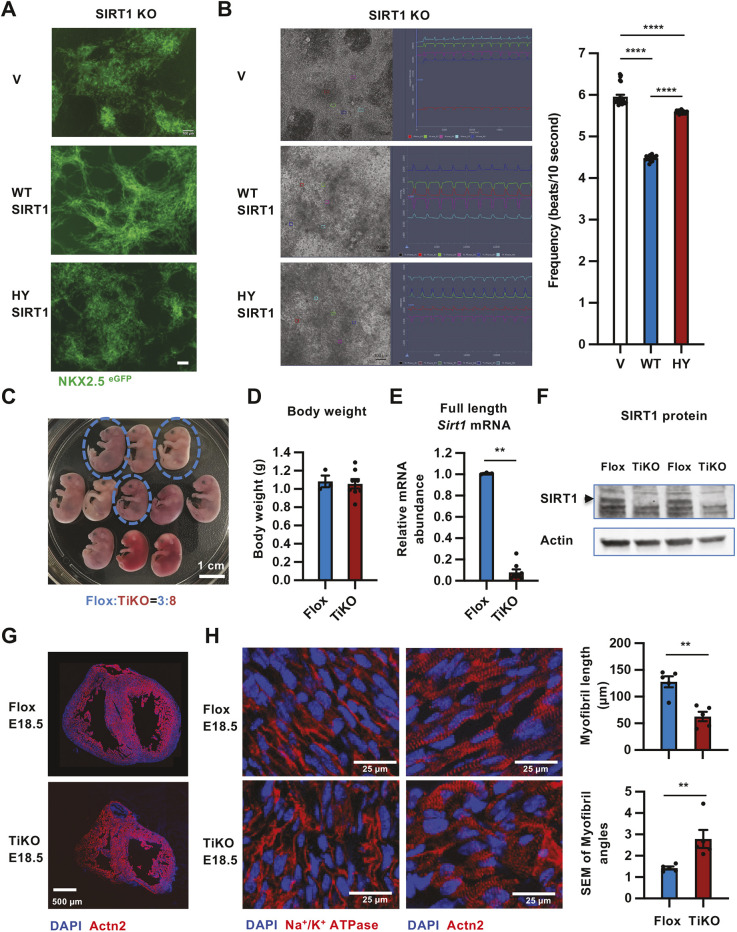
**SIRT1 regulates late-stage cardiomyocyte alignment *in vitro* and *in vivo*.** (A) Putting back WT, but not H363Y (HY) mutant, SIRT1 after differentiation of cardiomyocytes rescues the defective morphology of SIRT1 KO iCMs. Adeno-associated viruses (AAVs) containing empty vector (V), or construct expressing WT or HY SIRT1 proteins were transduced into SIRT1 KO iCMs as in Fig. S2A. The morphology of iCMs was analyzed 20 days after transduction. Scale bar: 100 µm. (B) Expression of WT SIRT1 after differentiation of cardiomyocytes reduces beating frequency of SIRT1 KO iCMs. SIRT1 KO iCMs were transduced with AAVs as in A. The beating frequencies were imaged and quantified in five independent fields from three biological replicates [*n*=25 (V), *n*=19 (WT) or *n*=17 (HY) different locations, ****P<0.0001, two-way ANOVA, values represent mean±s.e.m.]. (C,D) Deletion of SIRT1 from E12.5 does not affect overall survival and body weights of E18.5 embryos. Dams from Flox (female)×tamoxifen-inducible SIRT1 knockout (SIRT1 TiKO) (male) breeding pairs were dosed with tamoxifen from E12.5 to E15.5 to induce the deletion of *Sirt1* gene as described in the Materials and Methods and in Fig. S2B. Embryos were dissected on E18.5. Scale bar: 1 cm. (E) *Sirt1* mRNA levels in the brains of E18.5 Flox and TiKO embryos (*n*=3 for Flox, and *n*=8 for TiKO). (F) SIRT1 protein levels in the hearts of E18.5 Flox and TiKO embryos (*n*=2 pairs of Flox and TiKO). (G) Sections of hearts from Flox and SIRT1 TiKO E18.5 embryos. Hearts from E18.5 embryos were collected and stained with anti-ACTN2 antibodies. Scale bar: 500 µm. (H) Deletion of SIRT1 from E12.5 results in impaired cardiomyocyte and myofibril alignment as shown in Fig. S2C. The longitudinal sections localized to the middle of the left ventricle of hearts from E18.5 WT and TiKO embryos were stained with anti-Na^+^/K^+^ ATPase or anti-ACTN2 antibodies (*n*=5 individual embryos for each genotype); for the myofibril length analysis, a total of 80 myofibrils from WT embryos and 150 myofibrils from KO embryos were analyzed; for the myofibril alignment analysis, a total of 440 myofibril angles from WT embryos and 650 myofibril angles from KO embryos were analyzed; representative images are shown in C; **P<0.01, Mann-Whitney test, values represent mean±s.e.m.). Scale bars: 25 µm.

To further confirm our observations that SIRT1 primarily modulates cardiomyocyte alignment at late developmental stages but not differentiation of cardiomyocytes from cardiac progenitors, we first examined whether putting back SIRT1 after cardiomyocyte differentiation could rescue the defective morphology and beating patterns of SIRT1 KO iCMs *in vitro*. We induced the differentiation of SIRT1 KO mel1 cells to cardiomyocytes for 17 days, then transduced them with adeno-associated viruses (AAVs) expressing either WT SIRT1 or a catalytically inactive SIRT1 mutant [H363Y (HY) SIRT1, Fig. S2A]. As shown in Fig. 4A, compared to AAVs containing the empty vector (V), AAV-mediated expression of WT SIRT1 protein in SIRT1 KO iCMs significantly rescued their random distribution and misalignment 20 days after transduction. This morphological rescue was accompanied by a reduced beating frequency (Fig. 4B, WT SIRT1). In contrast, expression of the HY SIRT1 failed to improve morphological abnormality or only partially reduced the beating defects of SIRT1 KO iCMs (Fig. 4A,B, HY SIRT1), indicating that the deacetylase activity of SIRT1 is required for normal remodeling of iCMs.

We next examined whether deletion of SIRT1 at late developmental stages in mouse embryos, particularly after differentiation of embryonic cardiomyocytes, could lead to cardiac morphological alterations observed in the SIRT1 KO mice (Fig. 1). Mouse embryonic heart development includes overlapping phases of heart tube formation and chamber formation (de Boer et al., 2012). The initial heart tube formation starts around E7.5, when the cardiogenic part of the splanchnic mesoderm expressing NKX2.5 is present as bilateral plates of epithelium. The formation of the four-chambered heart starts around E9.5, with overall morphology in the ventricles and outflow tracts completed around E11 (de Boer et al., 2012). To induce the deletion of *Sirt1* gene at late developmental stages, we generated a tamoxifen-inducible SIRT1 knockout (SIRT1 TiKO) strain by breeding a *Sirt1* floxed allele (Cheng et al., 2003) with a tamoxifen-inducible Cre line (CAGGCre-ER^TM^ transgenic line from The Jackson Laboratory). We then gavaged dams from the Flox (female)×SIRT1 TiKO (male) breeding pairs with tamoxifen daily from E12.5 to E15.5, isolated dosed Flox and SIRT1 TiKO embryos at E18.5, and analyzed the morphology of their hearts (Fig. S2B). This maternal dosing strategy successfully deleted the *Sirt1* gene in SIRT1 TiKO embryos without any significant effects on embryo growth (Fig. 4C–F). Remarkably, deletion of SIRT1 at this late developmental stage was sufficient to reduce the size of the heart (Fig. 4G), and induce the irregular distribution of cardiomyocytes (Na^+^/K^+^ ATPase) and misalignment of myofibrils (ACTN2) *in vivo* (Fig. 4H; Fig. S2C). Therefore, SIRT1 regulates cardiomyocyte remodeling at late developmental stages both *in vitro* and *in vivo*.

### SIRT1 deficiency blunts the induction of chemotaxis in cardiomyocytes *in vitro* and *in vivo*

To gain molecular insights on how SIRT1 regulates cardiomyocyte alignment at late developmental stages, we analyzed the significantly altered Ingenuity Canonical Pathways that were enriched in the significantly altered gene list in E18.5 SIRT1 KO and WT mouse hearts (Table S2). As shown in Fig. S3A, the top six downregulated pathways in E18.5 SIRT1 KO embryonic hearts, as judged by their negative *z*-scores (Kramer et al., 2014), were all related to inflammation and chemotaxis signaling. In particular, the signaling of CXCR4, a widely expressed G-protein-coupled receptor critically involved in development and organogenesis (Busillo and Benovic, 2007), was significantly suppressed in E18.5 SIRT1 KO hearts (Fig. S3A,B). Interestingly, mice lacking CXCR4 or its chemokine ligand CXCL12 (SDF1) are perinatally lethal, along with intrauterine growth retardation and cardiac defects (Ma et al., 1998; Nagasawa et al., 1996; Zou et al., 1998). These phenotypes strikingly resemble the developmental defects observed in SIRT1 KO mice (Cheng et al., 2003; McBurney et al., 2003; Wang et al., 2008), suggesting that defective chemokine signaling, particularly CXCR4 signaling, may be responsible for defective cardiac phenotypes in SIRT1 KO embryos.

**Fig. 5.**
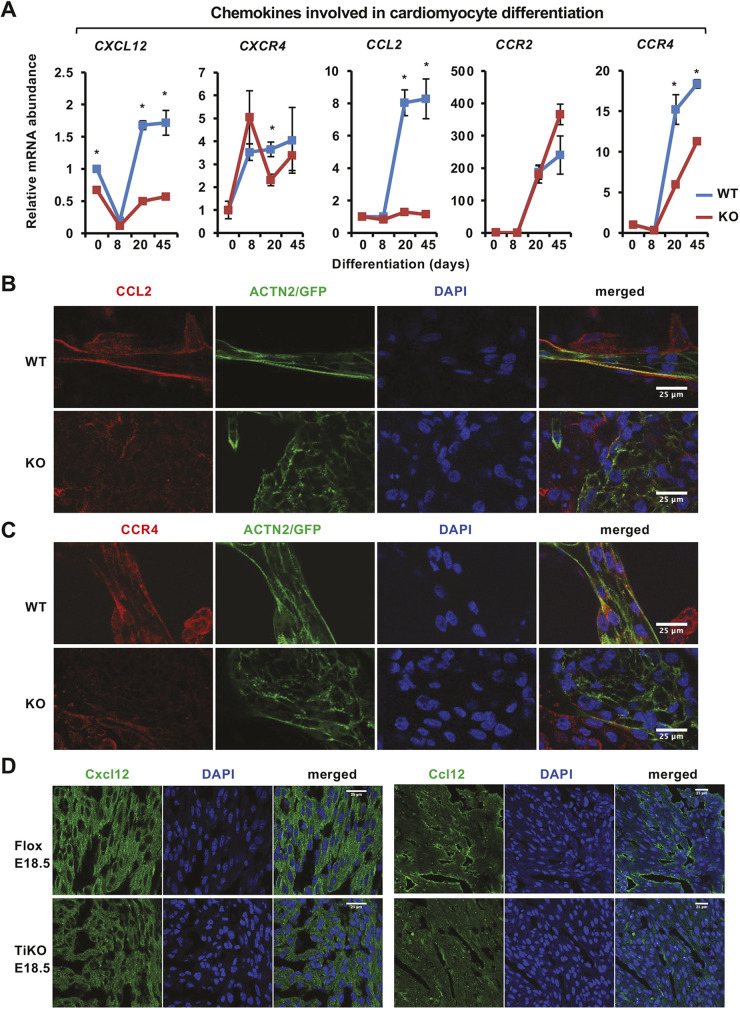
**SIRT1 deficiency blunts the induction of chemotaxis signaling in cardiomyocytes *in vitro* and *in vivo*.** (A) The induction of chemotaxis genes in WT and SIRT1 KO cardiomyocytes. WT and SIRT1 KO mel1 hESCs were induced for cardiomyocyte differentiation, and total RNA was collected on D0, D8, D20 and D45, and processed for qPCR analysis of *CCL2*, *CCR2*, *CCR4*, *CXCL12* and *CXCR4* (*n*=3 independent lines/genotype, *P<0.05, Student's *t*-test, values represent mean±s.e.m.). (B,C) The expression of CCL2 and CCR4 is reduced in SIRT1 KO iCMs. D20 WT and SIRT1 KO iCMs were fixed and stained with anti-CCL2 and anti-ACTN2 antibodies (B), or anti-CCR4 and anti-ACTN2 antibodies (C). Scale bars: 25 µm. (D) Deletion of SIRT1 from E12.5 embryos results in reduction of CXCL12 and CCL12 expression in cardiomyocytes. Heart sections from E18.5 Flox and TiKO embryos were stained with anti-CXCL12 (left panels) or anti-CCL12 (right panels) antibodies. Scale bars: 25 µm.

To test this possibility, we analyzed the expression of CXCR4 and its ligand CXCL12 during *in vitro* cardiomyocyte differentiation of WT and SIRT1 KO mel1 hESCs. *CXCR4* mRNA levels were quickly induced during the first 8 days of differentiation, then leveled off during the maturation stages in both WT and SIRT1 KO cells (Fig. 5A, CXCR4). The expression of *CXCL12* was reduced at D8 but then quickly re-induced and reached a plateau around D20 in WT cells. In SIRT1 KO cells, the induction of this chemokine was blunted (Fig. 5A, CXCL12), suggesting that the defective CXCR4 signaling detected in the transcriptome analysis (Fig. S3) is possibly due to defective CXCL12 production in SIRT1 KO cardiomyocytes.

Further survey of other chemokine signaling pathways revealed that the chemokine CCL2 (MCP-1) was dramatically induced around D20 during *in vitro* cardiomyocyte differentiation of WT mel1 hESCs, but this induction was almost completely blocked in SIRT1 KO cells (Fig. 5A, CCL2). CCL2 is a cytokine/chemokine that acts as a ligand for chemokine receptors CCR2 (Gschwandtner et al., 2019) and CCR4 (Zhang et al., 2006), displaying chemotactic activity for monocytes, basophils and T lymphocytes. CCL2 is one of the most highly expressed chemokines in human myocardium (Damas et al., 2000), and its expression has been recently detected in cardiomyocytes from human early-stage fetuses by single-cell RNA sequencing (Cui et al., 2019). CCR2, the main receptor of CCL2, exhibits mild expression in the human heart (Fig. S3C), and a single-nucleotide polymorphism of the *CCR2* gene is associated with myocardial infarction and heart failure, but not with coronary atherosclerosis, in human patients (Ortlepp et al., 2003). We confirmed that, during *in vitro* cardiomyocyte differentiation of hESCs, both *CCR2* and *CCR4* were massively induced at late stages, and the induction of *CCR4* was significantly reduced in SIRT1 KO cells (Fig. 5A, CCR2 and CCR4). Immunofluorescent analysis further indicated that, in WT D20 iCMs, both CCL2 and CCR4 were colocalized with or accompanied by ACTN2 on the cell membrane along the myofibrils (Fig. 5B,C, WT). In particular, CCL2 was abundant on the membrane of cells on the edge of a cell cluster, but exhibited low expression on the membrane of inner cells, indicating a certain degree of polarization of CCL2 (Fig. 5B). In contrast, CCL2 and CCR4 were reduced in expression, with decreased colocalization with ACTN2-positive myofibrils, in D20 SIRT1 KO iCMs compared to WT iCMs (Fig. 5B,C, KO). Therefore, the CCL2 signaling pathway is significantly suppressed and mislocalized in SIRT1-deficient iCMs.

Consistent with the observations in iCMs from hESCs, CXCL12 protein was widely expressed in the cardiomyocytes from Flox E18.5 hearts, yet its expression was markedly reduced in the cardiomyocytes from E18.5 SIRT1 KO hearts (Fig. 5D, left panels). Moreover, the mRNA expression of *Ccl12*, encoding a mouse chemokine closely related to human CCL2 (Jia et al., 1996) and a specific CCR2 ligand (Sarafi et al., 1997), was significantly reduced in E18.5 SIRT1 KO hearts compared to WT hearts (Fig. S3D). Interestingly, CCL12 protein in E18.5 Flox hearts was enriched in a subpopulation of cardiomyocytes, in which it was localized to striated sarcomere structures (Fig. 5D, right panels, Flox E18.5). Consistent with the mRNA levels from the E18.5 SIRT1 KO hearts, CCL12 protein expression was much lower in E18.5 SIRT1 TiKO cardiomyocytes than in Flox cardiomyocytes, although its expression may be strong in endocardial cells (Fig. 5D, right panels, TiKO E18.5). Taken together, our data demonstrate that SIRT1 deficiency results in defective chemokine signaling important for cardiac function *in vitro* and *in vivo*, which may be responsible for the cardiac defects observed in SIRT1-deficient iCMs and embryonic hearts.

### Inhibition of the CCL2 signaling pathway impairs cardiomyocyte alignment *in vitro*

**Fig. 6.**
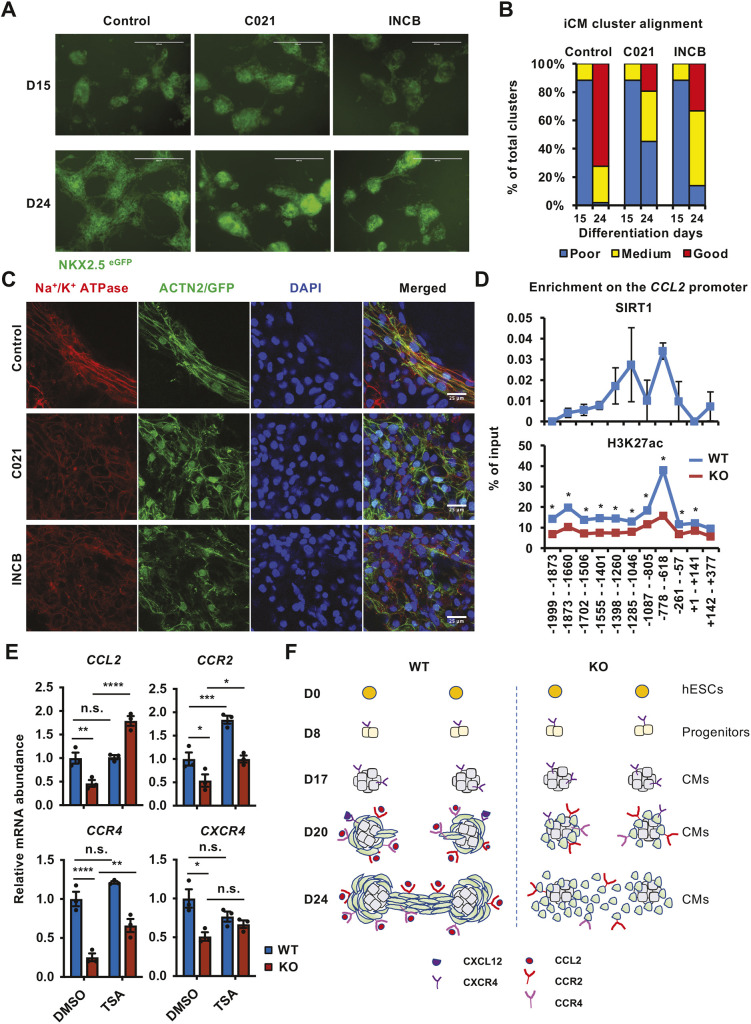
**Inhibition of CCL2 signaling impairs cardiomyocyte alignment.** (A) WT iCMs were treated with control, 2 µM CCR4 inhibitor C 021 dihydrochloride (C021) or 100 nM CCR2 inhibitor INCB 3284 dimesylate (INCB) from D15 to D24. The morphology of iCMs was analyzed by imaging of NKX2.5^eGFP^-positive cell clusters. Scale bars: 400 µm. (B) The alignments of cardiomyocyte clusters from WT and KO cells were categorized into three categories and quantified (*n*=17 images/fields for D15 control, C021 and INCB; *n*=51 images/field for D24 control, *n*=31 for D24 C021, and *n*=36 for D24 INCB). (C) WT iCMs treated as in A were stained with anti-ACTN2 and anti-Na^+^/K^+^ ATPase antibodies. Scale bars: 25 µm. (D) Deletion of SIRT1 reduces H3K27ac levels on the *CCL2* promoter in iCMs. D30 WT and SIRT1 KO iCMs were cross-linked, and the association of SIRT1 and H3K27ac on the *CCL2* promoter was analyzed by ChIP–qPCR as described in the Materials and Methods (*n*=3 biological repeats/genotype, *P<0.05, Student's *t*-test, values represent mean±s.e.m.). (E) Inhibition of HDACs by Trichostatin A (TSA) rescues the expression of genes in the CCL2 signaling pathway in SIRT1 KO iCMs. D30 WT and SIRT1 KO iCMs were treated with dimethyl sulfoxide or 0.6 µM TSA for 24 h. The expression of indicated genes was analyzed by qPCR (*n*=3 biological repeats/genotype; n.s., not significant; *P<0.05, **P<0.01, ***P<0.001, ****P<0.0001, two-way ANOVA, values represent mean±s.e.m.). (F) SIRT1 regulates cardiomyocyte remodeling during maturation.

The defective CXCL12/CXCR4 and CCL2/CCR2/CCR4 chemokine signaling in SIRT1 KO iCMs and mouse embryonic hearts (Fig. 5; Fig. S3) suggests that inhibition of these chemokine pathway may disrupt the alignment and maturation of iCMs. Because the CXCL12/CXCR4 pathway has already been established as an important signaling pathway for cardiac development (Ma et al., 1998; Nagasawa et al., 1996; Zou et al., 1998), we directly tested the CCL2 pathway. Indeed, when WT iCMs were treated with the CCR4 inhibitor C021 dihydrochloride (C021) or CCR2 inhibitor INCB 3284 dimesylate (INCB) from D15 to D24 after induction of differentiation, the alignment of NKX2.5^eGFP^-positive iCM clusters was impaired at D24 (Fig. 6A,B). Further immunofluorescent staining with anti-Na^+^/K^+^ ATPase and anti-ACTN2 antibodies revealed that pharmacological inhibition of CCL2 signaling by either C021 or INCB disrupted the alignment of myofibrils in iCMs (Fig. 6C). Therefore, CCL2 chemokine signaling is critically involved in the regulation of cardiomyocyte alignment and maturation *in vitro*.

To gain further understanding on how SIRT1 may regulate CCL2 chemokine signaling, we analyzed the association of SIRT1 protein with the promoter of the *CCL2* gene in WT iCMs by chromatin immunoprecipitation (ChIP). As shown in Fig. 6D, SIRT1 was enriched ∼600–800 bp upstream of the transcription start site of the *CCL2* gene (top), which was overlapped with a locus peaked for H3K27ac, an activation histone mark (bottom, WT). However, the enrichment of H3K27ac on this locus was significantly blunted in KO iCMs (Fig. 6D, bottom, KO), indicative of a strong attenuation of transcriptional activation of the *CCL2* gene in SIRT1 KO iCMs. Given that class I and II histone deacetylases (HDACs) could deacetylate H3K27ac to repress gene transcription and that SIRT1 inhibits HDAC1-dependent transcriptional repression via the RBP1 family (Binda et al., 2008), we hypothesized that SIRT1 may promote the expression of CCL2 by repressing HDAC1. Inhibition of HDAC1, therefore, could rescue CCL2 expression in SIRT1 KO cells. In line with this hypothesis, inhibition of class I and II HDACs by Trichostatin A (TSA) significantly reactivated the defective expression of the *CCL2* gene in SIRT1 KO, but not WT, iCMs (Fig. 6E, *CCL2*). TSA treatment also partially rescued the defective expression of *CCR2* and *CCR4* genes, particularly in the KO iCMs (Fig. 6E, *CCR2*, *CCR4*). However, the expression of *CXCR4* was not significantly modulated by TSA treatment in either WT or KO cells, indicating that this HDAC-dependent mechanism is specific to CCL2 signaling. Taken together, our results suggest a model in which SIRT1 promotes an open configuration of chromatin on the promoters of genes in the CCL2 signaling pathway, which in turn allows histone acetylation and transcriptional activation of these genes.

## DISCUSSION

Cardiomyocyte maturation and remodeling, including elongation and alignment, is important for cardiac function. Deciphering the underlying mechanisms would provide valuable insights to improve the integration efficiency of injected *in vitro* differentiated cardiomyocytes into the existing heart cells. However, how cardiomyocytes and myofibrils are aligned remains unclear, partially due to technical challenges in analyzing misalignment *in vivo* and in recapitulating remodeling events with isolated cardiomyocytes *in vitro*. In this study, utilizing genetically modified mouse models together with an *in vitro* differentiation system that induces differentiation and remodeling of cardiomyocytes, we dissected molecular mechanisms regulating cardiomyocyte/myofibril alignment and unexpectedly found that SIRT1 is a key mediator regulating cardiomyocyte/myofibril alignment (Fig. 6F).

SIRT1 has been implicated as an important regulator of heart development and normal cardiomyocyte functions (Chen et al., 2006; Cheng et al., 2003; Planavila et al., 2012; Sundaresan et al., 2011). However, the exact role of SIRT1 in this biological process remains unclear. Our present study provides evidence that SIRT1 primarily modulates cardiomyocyte/myofibril alignment and contraction during perinatal cardiomyocyte maturation, but with minimal effects on early cardiomyocyte fate specification/differentiation. For example, in the *in vitro* cardiomyocyte differentiation system, SIRT1 deletion does not block the differentiation of cardiac progenitors (Fig. 2). Consistently, SIRT1-deficient mouse hearts appeared to develop normally before E14.5 on the C57BL/6J background (data not shown), supporting the idea that SIRT1 is not required for specification of cardiac progenitors and cardiomyocytes. More importantly, putting back SIRT1 after D17 in the *in vitro* cardiomyocyte differentiation system is able to rescue morphological abnormality and beating defects of SIRT1 KO iCMs (Fig. 4A,B; Fig. S2A), while induced deletion of SIRT1 at the late developmental stages is sufficient to impair the normal distribution and alignment of cardiomyocytes/myofibrils (Fig. 4C–H; Fig. S2B,C).

Our study also identifies defective chemokine signaling as a possible underlying mechanism responsible for defective cardiac defects in SIRT1-deficient iCMs and mouse perinatal hearts. First, E18.5 SIRT1 KO mouse hearts are predominately defective in pathways involved in inflammation and CXCR4 chemokine signaling at the transcriptomic level (Fig. S3). Second, both CXCL12/CXCR4 and CCL2/CCR2/CCR4 pathways are induced during *in vitro* cardiomyocyte differentiation of hESCs, but SIRT1 deficiency dramatically blunts their induction (Fig. 5A). Third, we found that CCL2, CCR4 and CCL12 are colocalized with ACTN2 along the Z-line in iCMs and perinatal mouse hearts (Fig. 5B–D), as reported in a previous study on the localization of CXCR4 and CXCL12 protein (Segret et al., 2007). Deletion of SIRT1 in independent cell and mouse models disrupts sarcomere localization (Fig. 5B–D). Finally, and more importantly, pharmacological inhibition of CCL2 receptors impairs cardiomyocyte alignment *in vitro* (Fig. 6A–C). All these data indicate that the CCL2 pathway is important for cardiomyocyte alignment *in vitro*, and it is one of the underlying mechanisms by which SIRT1 promotes cardiomyocyte alignment. In immune cells, SIRT1 has a well-known activity in repression of inflammation and cell activation by directly deacetylating and inhibiting different transcription factors, including NF-κB (Yeung et al., 2004), AP-1 (Zhang et al., 2010), STAT3/Rorc (Limagne et al., 2017) and HIF-1α (Liu et al., 2014; Wang et al., 2016). Intriguingly, our data indicate that SIRT1 positively modulates chemokine signaling during cardiomyocyte remodeling and promotes cardiomyocyte alignment. Specifically, we show that SIRT1 enhances promoter histone acetylation levels and activates the expression of CCL2 by repressing HDACs (Fig. 6D,E), which are in line with previous reports that SIRT1 or deacetylation represses HDAC1 activity (Binda et al., 2008; Qiu et al., 2006). It is of great interest to further dissect the cell-type-specific regulation of chemokine signaling by SIRT1 in the future.

Our study has some limitations. First, we noticed that SIRT1 and the induced chemokines are expressed in cardiomyocytes, as well as many other cell types, in both mouse embryonic hearts and the *in vitro* cardiomyocyte differentiation system. Therefore, the universal manipulation of SIRT1/CCL2 signaling in our study did not allow us to dissect the cell-type-specific functions of these genes during cardiomyocyte remodeling. Future studies with cell-type-specific gene manipulations (e.g. cardiomyocyte-specific deletion of SIRT1) are required to further determine their specific role in cardiomyocytes. Second, most of our experiments, particularly the *in vitro* experiments, in this study have small sample sizes, which limits the statistical power of our data.

It is also worth noting that, although our present study suggests that the defective chemokine signaling may partially underlie SIRT1 deficiency-induced defects in cardiomyocyte alignment and maturation, SIRT1 deficiency is associated with a number of other transcriptional defects. For instance, the induction of TBX5 and GATA4, two key cardiogenesis transcription factors, was significantly reduced at the late stage of *in vitro* differentiation (Fig. 2C). The induction of *TNNT2*, *ACTN2*, *MYL3* and *TCAP*, genes encoding major sarcomere structural proteins or muscle contraction factors, was also mildly reduced at different differentiation time points *in vitro* (Fig. 2C). Additionally, cAMP signaling, NFAT signaling and calcium signaling were significantly upregulated in E18.5 SIRT1 KO hearts (Fig. S3A). It is possible that these alterations may also contribute to the misalignment and maturation phenotypes. Given that SIRT1 is capable of directly or indirectly regulating the acetylation and methylation levels of histones and transcription factors (Tang et al., 2017), it is likely that deletion of SIRT1 blunts the early chromatin remodeling events required for transcription of factors involved in the above signaling pathways. Future studies are needed to dissect the molecular mechanisms underlying this transcriptional regulation.

In summary, we have shown that SIRT1 plays a vital role in the regulation of myofibril alignment and cardiomyocyte maturation during animal development. Our study highlights the therapeutic potential of SIRT1-activating compounds in the treatment of human heart disease.

## MATERIALS AND METHODS

### Animal studies

Whole-body germ-line SIRT1 knockout (*Sirt1*^−/−^) mice on the C57BL/6J background have been reported previously (Tang et al., 2014). To investigate the heart developmental phenotypes of SIRT1 KO embryos, 2- to 3-month-old SIRT1 heterozygous female mice were bred with age-matched heterozygous male mice. The following morning, females with the mating plug (E0.5) were separated from the male into a new cage. Conceptus/embryos at E14.5, E18.5 and postnatal day (P)0 (newborn) were collected and analyzed. Hearts from both sexes were analyzed.

The SIRT1 TiKO strain was generated by breeding the *Sirt1* floxed allele (Cheng et al., 2003) with a tamoxifen-inducible Cre line in which the expression of the CreER^TM^ fusion protein is driven by the chicken beta actin promoter/enhancer coupled with the cytomegalovirus (CMV) immediate-early enhancer (CAGGCre-ER^TM^ transgenic line, The Jackson Laboratory, stock number 004682). To induce the deletion of the *Sirt1* gene at late developmental stages, dams from the Flox (*Sirt1^flox/flox^, CreER^TM^−*, female)×SIRT1 TiKO (*Sirt1^flox/flox^, CreER^TM^+*, male) breeding pairs were gavaged with 100 mg/kg body weight of tamoxifen (in corn oil) daily from E12.5 to E15.5. Flox and SIRT1 TiKO embryos were then dissected at E18.5 from the dosed dams, and their hearts were collected and analyzed. Hearts from both sexes were analyzed.

All conducted animal experiments were approved by the National Institute of Environmental Health Sciences (NIEHS)/National institutes of Health (NIH) Animal Care and Use Committee.

### Antibodies

Anti-SIRT1 antibodies [human specific: 2493, 1:1000 dilution for western blotting (WB); mouse specific: 2028, 1:1000 dilution for WB] were from Cell Signaling Technology. Anti-ACTN2 antibodies [ab9465, 1:50 dilution for immunofluorescence (IF); ab68167, 1:1000 dilution for WB and 1:200 for IF] and anti-Na^+^/K^+^ ATPase antibody (ab76020, 1:200 dilution for IF) were from Abcam. Anti-Actin antibody was from Millipore (MAB1501, 1:10000 dilution for WB). Anti-CCL2 antibody (MABN712; 1:200 dilution for IF) was from Sigma, anti-CCR4 antibody (MAB1567; 1:200 dilution for IF) was from R&D Systems, and anti-CCL12 antibody (abx103190; 1:200 dilution for IF) was from Abbexa. In ChIP analysis, 3 μl anti-histone H3 (acetyl K27) antibody (Abcam, ab4729) was used per reaction.

### Microarray analysis

Total RNAs from WT and SIRT1 KO mouse E18.5 hearts were isolated with the TRIzol reagent, followed by RNA clean-up using a Qiagen RNeasy mini-kit with on-column DNAse I treatment. Gene expression analysis was conducted using Agilent Whole Human Genome 4×44 multiplex format oligo arrays (014850, for RNAs from mel1 cells) or Agilent Whole Mouse Genome 4×44 multiplex format oligo arrays (014868, for RNAs from mouse E18.5 hearts) (Agilent Technologies) following the Agilent one-color microarray-based gene expression analysis protocol. Starting with 500 ng total RNA, Cy3-labeled cRNA was produced according to the manufacturer's protocol. For each sample, 1.65 μg Cy3-labeled cRNAs were fragmented and hybridized for 17 h in a rotating hybridization oven. Slides were washed and then scanned with an Agilent Scanner. Data were obtained using Agilent Feature Extraction Software (v9.5), using the one-color defaults for all parameters. The Agilent Feature Extraction Software performed error modeling, adjusting for additive and multiplicative noise. Differential gene expression was examined using Partek Genomics Suite (Partek, St Louis, MO, USA). To identify differentially expressed probes, analysis of variance (ANOVA), Benjamini–Hochberg multiple test correction was used (false discovery rate<0.05). Partek Genomics Suite was further used to generate heat maps for visual analyses and to support generation of hierarchical clustering dendrograms. Lists of significant probes were further analyzed using IPA (Content version 26127183, Ingenuity Systems, Redwood City, CA, USA).

### Electron microscopy procedure

To compare the morphological difference between WT and SIRT1 KO hearts, heart tissues dissected from WT and SIRT1 KO E18.5 mice were fixed with Trumps 4F:1G fixative overnight at 4°C, processed with a Leica EM TP processor, rinsed with phosphate buffer and post-fixed in 1% osmium tetroxide in phosphate buffer. The post-fixed tissues were then rinsed in water, dehydrated in an ethanolic series culminating in acetone and further infiltrated with Poly/Bed 812 epoxide resin. After polymerization, the resulting blocks were trimmed, and semithin sections (∼0.5 µm thick) were cut, mounted on glass slides and stained with 1% Toluidine Blue O in 1% sodium borate prior to being examined with a light microscope. After inspection with light microscopy, ultrathin sections (80–90 nm thick) were subsequently cut, placed onto 200-mesh copper grids, and then stained with aqueous uranyl acetate and lead citrate. Digital images were captured with a Gatan Orius SC1000/SC600 attached to a FEICO Tecnai T120 transmission electron microscope. The width of the Z-line and the angle of individual myofibrils were measured from five individual embryos with a total of 35 observation fields/genotype. The degrees of myofibril alignment of WT and SIRT1 KO mouse cardiomyocytes were compared using the s.e.m. of the angles of individual myofibrils against the horizontal edge of the images.

### Cell culture

NKX2.5^eGFP/w^ mel1 hESCs were obtained with permission from Dr Andrew Elefanty and Dr Edouard Stanley at the University of Queensland, Australia (NIH registration number 0139), and cultured and maintained on Matrigel (Corning) in TeSR-E^8^ medium (StemCells Inc.).

### *In vitro* cardiomyocyte differentiation and fluorescence-activated cell sorting

To induce cardiomyocytes, WT and SIRT1 KO NKX2.5^eGFP/w^ mel1 hESCs generated by CRISPR/Cas9 gene-editing technology were cultured with 3–5 µM CHIR-99021 (Selleckchem) for 3 days, then switched and incubated with 5 µM IWR (Selleckchem) for 2 days in B27 minus insulin medium (Life Technologies). After washing, cells were cultured in B27 minus insulin medium for 2 more days, and thereafter maintained in B27 medium. To sort eGFP+ D17 cardiomyocytes, cells were trypsinized and resuspended with PBS. After being centrifuged at 300 ***g*** for 3 minutes, cell pellets were resuspended with 1 ml PBS and eGFP+ cells were sorted and collected by a flow cytometer.

### Morphological analyses of cardiomyocytes by light microscopy

To compare the morphological difference in WT and SIRT1 KO iCMs (Fig. 3E,F), the sarcomeres were immunostained with an antibody against ACTN2, and the lengths of myofibrils from three independent experiments were quantified and compared. To quantify and compare the degrees of myofibril alignment between WT and SIRT1 KO iCMs, the angles of individual myofibrils against the horizontal edge of the images were measured and the s.e.m. of the angles calculated.

### ChIP analysis

To analyze the occupancy of SIRT1 and H3K27ac on the promoter of the *CCL2* gene, 1×10^7^ WT or SIRT1-deficient cells, 30 days after induction from mel1 hESCs, were cross-linked, harvested and sonicated. The resultant sonicated chromatin was processed for immunoprecipitation with 5 µl anti-SIRT1 (Cell Signaling Technology, #2493) antibody or 3 µl anti-H3K27ac (Abcam, ab177178) antibody. The DNA fragments from each sample were then analyzed by qPCR assay with primers covering −1999 to+377 bp of the human *CCL2* promoter.

### Statistics

Values are expressed as mean±s.e.m. from at least three independent experiments or biological replicates, unless otherwise indicated in the figure legend. Significant differences between means with two comparison groups were analyzed by two-tailed, unpaired, non-parametric Mann–Whitney test (animal experiments) or two-tailed, unpaired Student's *t*-test (cell culture experiments). Significant differences between means with more than two comparison groups were analyzed by two-way ANOVA with adjustments on multiple comparisons. For all comparisons, differences were considered significant at *P*<0.05. Statistical analyses were performed using either Microsoft Excel or Prism.

Statistical analyses for microarray data are detailed in the ‘Microarray analysis’ section.

## Supplementary Material

10.1242/joces.259076_sup1 Supplementary information

## References

[JCS259076C1] Binda, O., Nassif, C. and Branton, P. E. (2008). SIRT1 negatively regulates HDAC1-dependent transcriptional repression by the RBP1 family of proteins. *Oncogene* 27, 3384-3392. 10.1038/sj.onc.1211014 18193082

[JCS259076C2] Busillo, J. M. and Benovic, J. L. (2007). Regulation of CXCR4 signaling. *Biochim. Biophys. Acta* 1768, 952-963. 17169327 10.1016/j.bbamem.2006.11.002 PMC1952230

[JCS259076C3] Chen, I. Y., Lypowy, J., Pain, J., Sayed, D., Grinberg, S., Alcendor, R. R., Sadoshima, J. and Abdellatif, M. (2006). Histone H2A.z is essential for cardiac myocyte hypertrophy but opposed by silent information regulator 2alpha. *J. Biol. Chem.* 281, 19369-19377. 10.1074/jbc.M601443200 16687393

[JCS259076C4] Cheng, H. L., Mostoslavsky, R., Saito, S., Manis, J. P., Gu, Y., Patel, P., Bronson, R., Appella, E., Alt, F. W. and Chua, K. F. (2003). Developmental defects and p53 hyperacetylation in Sir2 homolog (SIRT1)-deficient mice. *Proc. Natl. Acad. Sci. USA* 100, 10794-10799. 10.1073/pnas.1934713100 12960381 PMC196882

[JCS259076C5] Cui, Y., Zheng, Y., Liu, X., Yan, L., Fan, X., Yong, J., Hu, Y., Dong, J., Li, Q., Wu, X. et al. (2019). Single-cell transcriptome analysis maps the developmental track of the human heart. *Cell Rep.* 26, 1934-1950. 30759401 10.1016/j.celrep.2019.01.079

[JCS259076C6] Damas, J. K., Eiken, H. G., Oie, E., Bjerkeli, V., Yndestad, A., Ueland, T., Tonnessen, T., Geiran, O. R., Aass, H., Simonsen, S. et al. (2000). Myocardial expression of CC- and CXC-chemokines and their receptors in human end-stage heart failure. *Cardiovasc. Res.* 47, 778-787. 10.1016/S0008-6363(00)00142-5 10974226

[JCS259076C7] de Boer, B. A., van den Berg, G., de Boer, P. A., Moorman, A. F. and Ruijter, J. M. (2012). Growth of the developing mouse heart: an interactive qualitative and quantitative 3D atlas. *Dev. Biol.* 368, 203-213. 10.1016/j.ydbio.2012.05.001 22617458

[JCS259076C8] Di Maio, A., Karko, K., Snopko, R. M., Mejia-Alvarez, R. and Franzini-Armstrong, C. (2007). T-tubule formation in cardiacmyocytes: two possible mechanisms? *J. Muscle Res. Cell Motil.* 28, 231-241. 10.1007/s10974-007-9121-x 17940841

[JCS259076C9] Elliott, D. A., Braam, S. R., Koutsis, K., Ng, E. S., Jenny, R., Lagerqvist, E. L., Biben, C., Hatzistavrou, T., Hirst, C. E., Yu, Q. C. et al. (2011). NKX2-5(eGFP/w) hESCs for isolation of human cardiac progenitors and cardiomyocytes. *Nat. Methods* 8, 1037-1040. 10.1038/nmeth.1740 22020065

[JCS259076C10] Fan, W., Tang, S., Fan, X., Fang, Y., Xu, X., Li, L., Xu, J., Li, J. L., Wang, Z. and Li, X. (2021). SIRT1 regulates sphingolipid metabolism and neural differentiation of mouse embryonic stem cells through c-Myc- SMPDL3B. *Elife* 10, e67452. 34042046 10.7554/eLife.67452 PMC8216717

[JCS259076C11] Gschwandtner, M., Derler, R. and Midwood, K. S. (2019). More than just attractive: how CCL2 influences myeloid cell behavior beyond chemotaxis. *Front. Immunol.* 10, 2759. 10.3389/fimmu.2019.02759 31921102 PMC6923224

[JCS259076C12] Han, M. K., Song, E. K., Guo, Y., Ou, X., Mantel, C. and Broxmeyer, H. E. (2008). SIRT1 regulates apoptosis and Nanog expression in mouse embryonic stem cells by controlling p53 subcellular localization. *Cell Stem Cell* 2, 241-251. 10.1016/j.stem.2008.01.002 18371449 PMC2819008

[JCS259076C13] Hirschy, A., Schatzmann, F., Ehler, E. and Perriard, J. C. (2006). Establishment of cardiac cytoarchitecture in the developing mouse heart. *Dev. Biol.* 289, 430-441. 10.1016/j.ydbio.2005.10.046 16337936

[JCS259076C14] Houtkooper, R. H., Pirinen, E. and Auwerx, J. (2012). Sirtuins as regulators of metabolism and healthspan. *Nat. Rev. Mol. Cell Biol.* 13, 225-238. 10.1038/nrm3293 22395773 PMC4872805

[JCS259076C15] Imai, S. and Guarente, L. (2010). Ten years of NAD-dependent SIR2 family deacetylases: implications for metabolic diseases. *Trends Pharmacol. Sci.* 31, 212-220. 10.1016/j.tips.2010.02.003 20226541 PMC3526941

[JCS259076C16] Jia, G. Q., Gonzalo, J. A., Lloyd, C., Kremer, L., Lu, L., Martinez, A. C., Wershil, B. K. and Gutierrez-Ramos, J. C. (1996). Distinct expression and function of the novel mouse chemokine monocyte chemotactic protein-5 in lung allergic inflammation. *J. Exp. Med.* 184, 1939-1951. 10.1084/jem.184.5.1939 8920881 PMC2192876

[JCS259076C17] Kramer, A., Green, J., Pollard, J., Jr. and Tugendreich, S. (2014). Causal analysis approaches in Ingenuity Pathway Analysis. *Bioinformatics* 30, 523-530. 10.1093/bioinformatics/btt703 24336805 PMC3928520

[JCS259076C18] Lian, X., Hsiao, C., Wilson, G., Zhu, K., Hazeltine, L. B., Azarin, S. M., Raval, K. K., Zhang, J., Kamp, T. J. and Palecek, S. P. (2012). Robust cardiomyocyte differentiation from human pluripotent stem cells via temporal modulation of canonical Wnt signaling. *Proc. Natl. Acad. Sci. USA* 109, E1848-E1857. 10.1073/pnas.1200250109 22645348 PMC3390875

[JCS259076C19] Lian, X., Zhang, J., Azarin, S. M., Zhu, K., Hazeltine, L. B., Bao, X., Hsiao, C., Kamp, T. J. and Palecek, S. P. (2013). Directed cardiomyocyte differentiation from human pluripotent stem cells by modulating Wnt/beta-catenin signaling under fully defined conditions. *Nat. Protoc.* 8, 162-175. 10.1038/nprot.2012.150 23257984 PMC3612968

[JCS259076C20] Limagne, E., Thibaudin, M., Euvrard, R., Berger, H., Chalons, P., Vegan, F., Humblin, E., Boidot, R., Rebe, C., Derangere, V. et al. (2017). Sirtuin-1 activation controls tumor growth by impeding Th17 differentiation via STAT3 deacetylation. *Cell Rep.* 19, 746-759. 10.1016/j.celrep.2017.04.004 28445726

[JCS259076C21] Liu, G., Bi, Y., Shen, B., Yang, H., Zhang, Y., Wang, X., Liu, H., Lu, Y., Liao, J., Chen, X. et al. (2014). SIRT1 limits the function and fate of myeloid-derived suppressor cells in tumors by orchestrating HIF-1alpha-dependent glycolysis. *Cancer Res.* 74, 727-737. 10.1158/0008-5472.CAN-13-2584 24351289

[JCS259076C22] Ma, Q., Jones, D., Borghesani, P. R., Segal, R. A., Nagasawa, T., Kishimoto, T., Bronson, R. T. and Springer, T. A. (1998). Impaired B-lymphopoiesis, myelopoiesis, and derailed cerebellar neuron migration in CXCR4- and SDF-1-deficient mice. *Proc. Natl. Acad. Sci. USA* 95, 9448-9453. 10.1073/pnas.95.16.9448 9689100 PMC21358

[JCS259076C23] McBurney, M. W., Yang, X., Jardine, K., Hixon, M., Boekelheide, K., Webb, J. R., Lansdorp, P. M. and Lemieux, M. (2003). The mammalian SIR2alpha protein has a role in embryogenesis and gametogenesis. *Mol. Cell. Biol.* 23, 38-54. 10.1128/MCB.23.1.38-54.2003 12482959 PMC140671

[JCS259076C24] McCain, M. L. and Parker, K. K. (2011). Mechanotransduction: the role of mechanical stress, myocyte shape, and cytoskeletal architecture on cardiac function. *Pflugers Arch.* 462, 89-104. 10.1007/s00424-011-0951-4 21499986

[JCS259076C25] Nagasawa, T., Hirota, S., Tachibana, K., Takakura, N., Nishikawa, S., Kitamura, Y., Yoshida, N., Kikutani, H. and Kishimoto, T. (1996). Defects of B-cell lymphopoiesis and bone-marrow myelopoiesis in mice lacking the CXC chemokine PBSF/SDF-1. *Nature* 382, 635-638. 10.1038/382635a0 8757135

[JCS259076C26] Ortlepp, J. R., Vesper, K., Mevissen, V., Schmitz, F., Janssens, U., Franke, A., Hanrath, P., Weber, C., Zerres, K. and Hoffmann, R. (2003). Chemokine receptor (CCR2) genotype is associated with myocardial infarction and heart failure in patients under 65 years of age. *J. Mol. Med. (Berl.)* 81, 363-367. 10.1007/s00109-003-0435-x 12719858

[JCS259076C27] Planavila, A., Dominguez, E., Navarro, M., Vinciguerra, M., Iglesias, R., Giralt, M., Lope-Piedrafita, S., Ruberte, J. and Villarroya, F. (2012). Dilated cardiomyopathy and mitochondrial dysfunction in Sirt1-deficient mice: a role for Sirt1-Mef2 in adult heart. *J. Mol. Cell. Cardiol.* 53, 521-531. 10.1016/j.yjmcc.2012.07.019 22986367

[JCS259076C28] Qiu, Y., Zhao, Y., Becker, M., John, S., Parekh, B. S., Huang, S., Hendarwanto, A., Martinez, E. D., Chen, Y., Lu, H. et al. (2006). HDAC1 acetylation is linked to progressive modulation of steroid receptor-induced gene transcription. *Mol. Cell* 22, 669-679. 10.1016/j.molcel.2006.04.019 16762839

[JCS259076C29] Sarafi, M. N., Garcia-Zepeda, E. A., MacLean, J. A., Charo, I. F. and Luster, A. D. (1997). Murine monocyte chemoattractant protein (MCP)-5: a novel CC chemokine that is a structural and functional homologue of human MCP-1. *J. Exp. Med.* 185, 99-109. 10.1084/jem.185.1.99 8996246 PMC2196097

[JCS259076C30] Segret, A., Rucker-Martin, C., Pavoine, C., Flavigny, J., Deroubaix, E., Chatel, M. A., Lombet, A. and Renaud, J. F. (2007). Structural localization and expression of CXCL12 and CXCR4 in rat heart and isolated cardiac myocytes. *J. Histochem. Cytochem.* 55, 141-150. 10.1369/jhc.6A7050.2006 17046839

[JCS259076C31] Sundaresan, N. R., Pillai, V. B. and Gupta, M. P. (2011). Emerging roles of SIRT1 deacetylase in regulating cardiomyocyte survival and hypertrophy. *J. Mol. Cell. Cardiol.* 51, 614-618. 10.1016/j.yjmcc.2011.01.008 21276800 PMC3442925

[JCS259076C32] Tang, S., Huang, G., Fan, W., Chen, Y., Ward, J. M., Xu, X., Xu, Q., Kang, A., McBurney, M. W., Fargo, D. C. et al. (2014). SIRT1-mediated deacetylation of CRABPII regulates cellular retinoic acid signaling and modulates embryonic stem cell differentiation. *Mol. Cell* 55, 843-855. 10.1016/j.molcel.2014.07.011 25155613 PMC4228935

[JCS259076C33] Tang, S., Fang, Y., Huang, G., Xu, X., Padilla-Banks, E., Fan, W., Xu, Q., Sanderson, S. M., Foley, J. F., Dowdy, S. et al. (2017). Methionine metabolism is essential for SIRT1-regulated mouse embryonic stem cell maintenance and embryonic development. *EMBO J.* 36, 3175-3193. 10.15252/embj.201796708 29021282 PMC5666621

[JCS259076C34] Wang, Y., Bi, Y., Chen, X., Li, C., Li, Y., Zhang, Z., Wang, J., Lu, Y., Yu, Q., Su, H. et al. (2016). Histone deacetylase SIRT1 negatively regulates the differentiation of interleukin-9-producing CD4(+) T cells. *Immunity* 44, 1337-1349. 10.1016/j.immuni.2016.05.009 27317260

[JCS259076C35] Wang, R. H., Sengupta, K., Li, C., Kim, H. S., Cao, L., Xiao, C., Kim, S., Xu, X., Zheng, Y., Chilton, B. et al. (2008). Impaired DNA damage response, genome instability, and tumorigenesis in SIRT1 mutant mice. *Cancer Cell* 14, 312-323. 10.1016/j.ccr.2008.09.001 18835033 PMC2643030

[JCS259076C36] Yeung, F., Hoberg, J. E., Ramsey, C. S., Keller, M. D., Jones, D. R., Frye, R. A. and Mayo, M. W. (2004). Modulation of NF-kappaB-dependent transcription and cell survival by the SIRT1 deacetylase. *EMBO J.* 23, 2369-2380. 10.1038/sj.emboj.7600244 15152190 PMC423286

[JCS259076C37] Zhang, T., Somasundaram, R., Berencsi, K., Caputo, L., Gimotty, P., Rani, P., Guerry, D., Swoboda, R. and Herlyn, D. (2006). Migration of cytotoxic T lymphocytes toward melanoma cells in three-dimensional organotypic culture is dependent on CCL2 and CCR4. *Eur. J. Immunol.* 36, 457-467. 10.1002/eji.200526208 16421945

[JCS259076C38] Zhang, R., Chen, H. Z., Liu, J. J., Jia, Y. Y., Zhang, Z. Q., Yang, R. F., Zhang, Y., Xu, J., Wei, Y. S., Liu, D. P. et al. (2010). SIRT1 suppresses activator protein-1 transcriptional activity and cyclooxygenase-2 expression in macrophages. *J. Biol. Chem.* 285, 7097-7110. 10.1074/jbc.M109.038604 20042607 PMC2844159

[JCS259076C39] Ziman, A. P., Gomez-Viquez, N. L., Bloch, R. J. and Lederer, W. J. (2010). Excitation-contraction coupling changes during postnatal cardiac development. *J. Mol. Cell. Cardiol.* 48, 379-386. 10.1016/j.yjmcc.2009.09.016 19818794 PMC3097073

[JCS259076C40] Zou, Y. R., Kottmann, A. H., Kuroda, M., Taniuchi, I. and Littman, D. R. (1998). Function of the chemokine receptor CXCR4 in haematopoiesis and in cerebellar development. *Nature* 393, 595-599. 10.1038/31269 9634238

