# Supplementary material for: SIRT1 regulates cardiomyocyte alignment during maturation
Source: J Cell Sci. 2022 Apr 1;135(7):jcs259076. doi: 10.1242/jcs.259076 (PMC9016619; doi:10.1242/jcs.259076)
Supplement: Supplementary information [file joces-135-259076-s1.pdf]

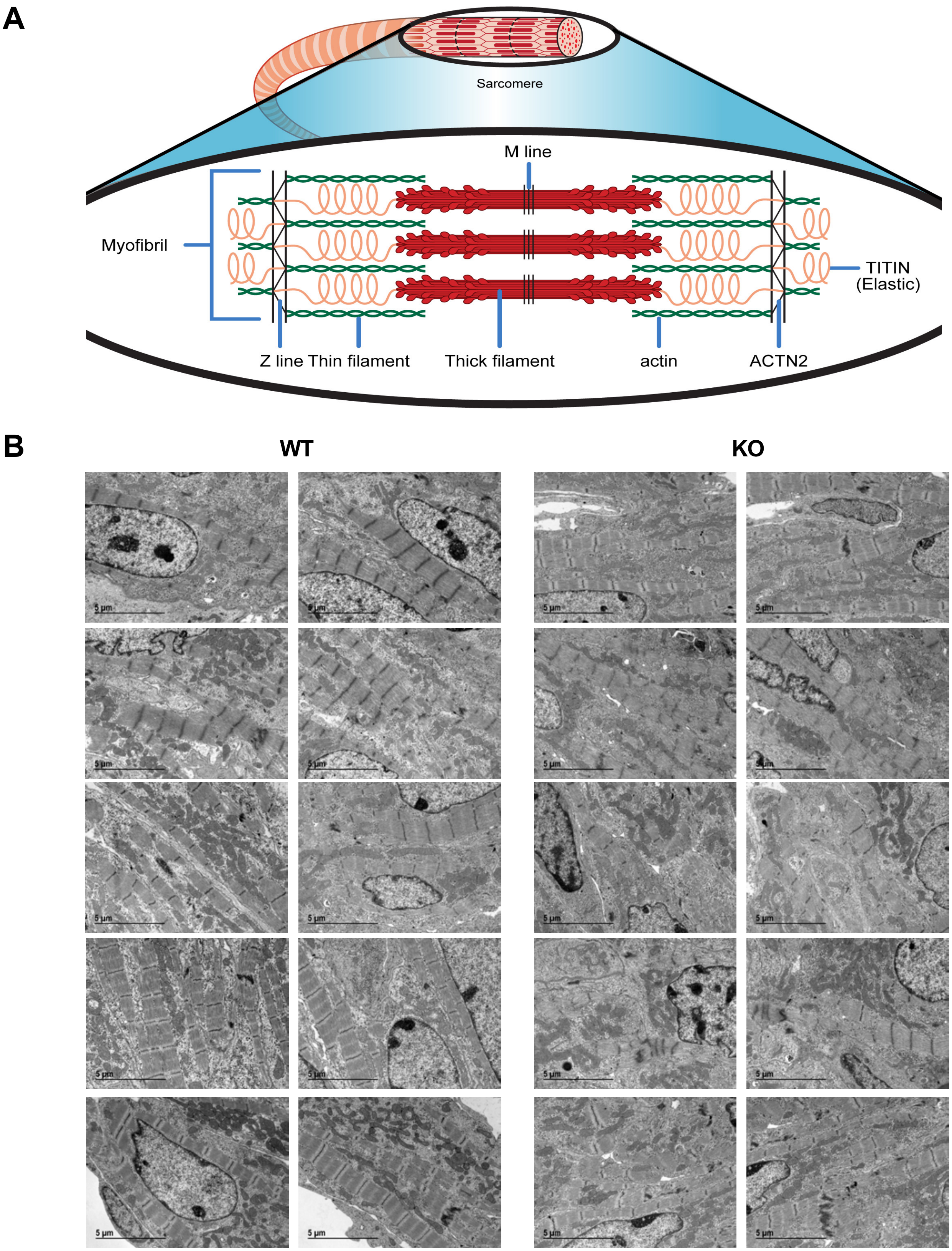

Fig. S1. SIRT1 KO mouse hearts have altered sarcomere ultrastructures.

(A) A cartoon model of sarcomere indicating the relative positions of Z-line, ACTN2, TITIN, and actin (thin) and myosin (thick) filaments. (B) Hearts from WT and SIRT1 KO embryos at E18.5 day were fixed and processed for electron microscopy analysis of sarcomere structures as described in Methods (n=5 individual embryos/group, and 2 representative images/embryo were shown).

Journal of Cell Science • Supplementary information

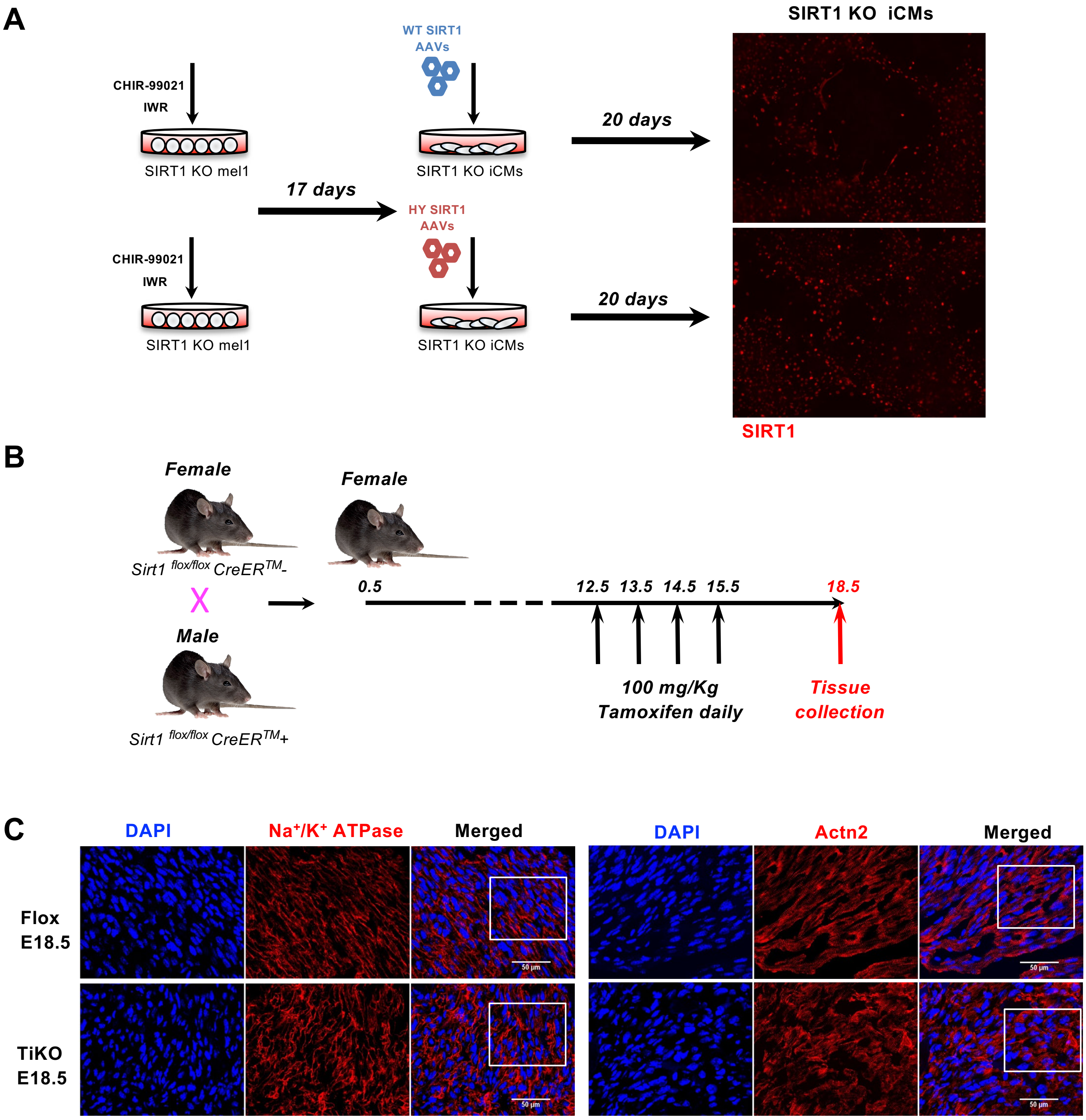

Fig. S2. SIRT1 regulates late-stage cardiomyocyte alignment in vitro and in vivo.

(A) Schematic representation of the strategy to express WT or HY mutant SIRT1 after differentiation of SIRT1 KO iCMs. AAVs expressing WT or SIRT1 HY mutant proteins were transduced into SIRT1 KO iCMs at D17 after induction of iCM differentiation. The expression of SIRT1 was analyzed 20 days after transduction. (B) Schematic representation of the strategy to induce SIRT1 deletion at late embryonic stages by maternal oral gavage of tamoxifen. (C) Deletion of SIRT1 from E12.5 results in impaired cardiomyocyte and myofibril alignment. Heart sections from E18.5 WT and TiKO embryos were stained with anti-Na+/K+ ATPase or anti-Actn2 antibodies. Scale bar: 50 µm.

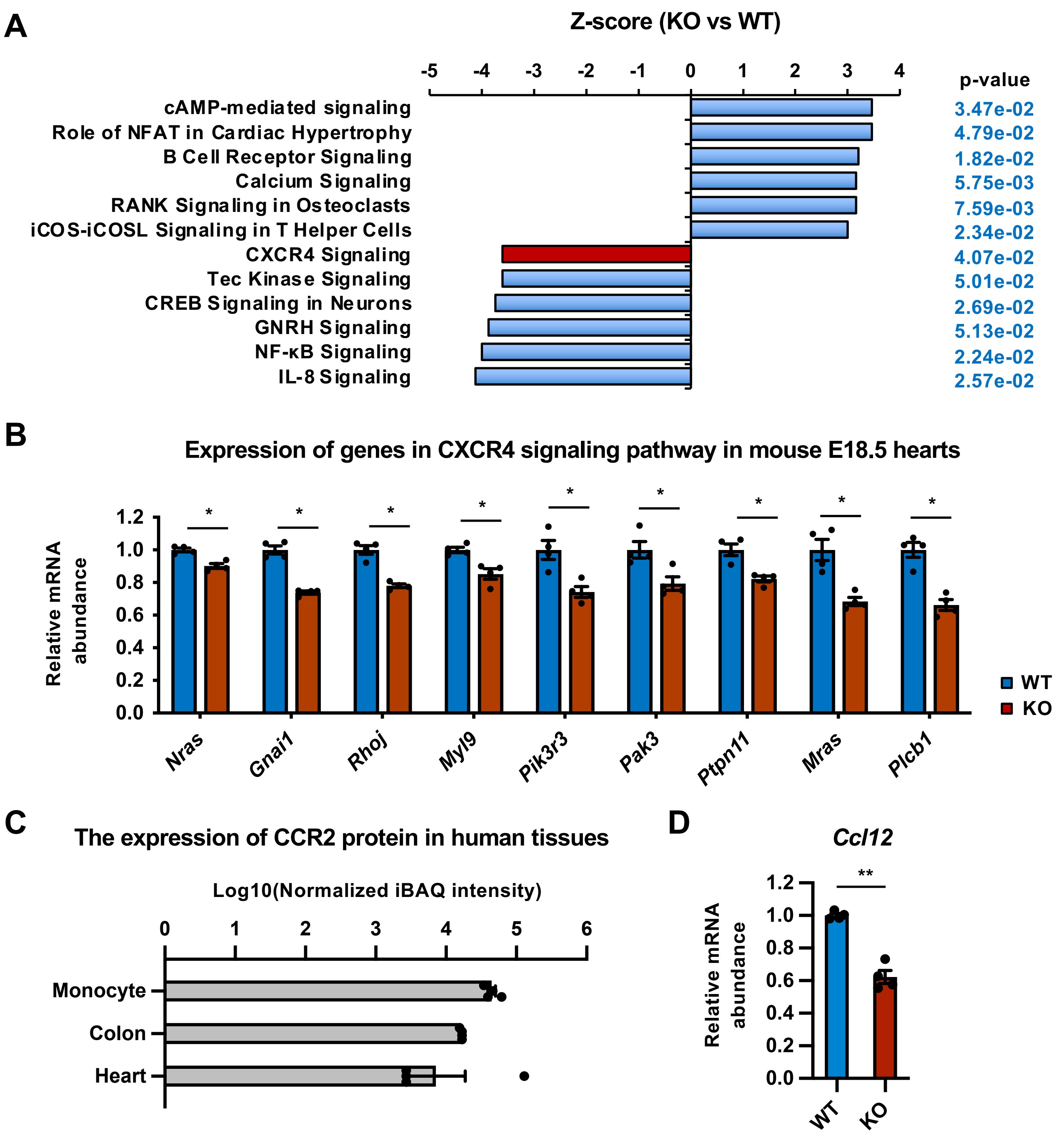

Fig. S3. Chemotaxis is important for heart development.

(A-B) SIRT1 KO E18.5 mouse hearts are defective in expression of genes involved in inflammation and chemotaxis. (A) The top canonical pathways enriched in the differentially expressed gene list from SIRT1 KO vs WT E18.5 mouse hearts. The significantly altered genes in WT and SIRT1 KO E18.5 mouse hearts were analyzed by microarray analysis and the enrich pathways were identified by the IPA analysis. Negative Z-score is indicative of downregulated pathways in SIRT1 KO E18.5 mouse hearts, and positive Z-score is indicative of upregulated pathways in SIRT1 KO E18.5 mouse hearts (n=4 embryos/genotype). (B) The expression of genes involved in CXCR4 signaling pathway is reduced in SIRT1 KO E18.5 mouse hearts. The relative mRNA levels of indicated genes were determined by microarray analysis (n=4 embryos/genotype, *p<0.05, values represent Mean ± SEM). (C) CCR2 is expressed in human heart. The median protein expression of CCR2 in indicated human tissues/cells was obtained from Proteomics DB (https://www.proteomicsdb.org) (n=4 for each tissue). (D) The mRNA levels of Ccl12 are significantly reduced in E18.5 SIRT1 KO mouse hearts. The relative mRNA levels of Ccl12 were determined by microarray analysis (n=4 embryos/genotype, **p<0.01, values represent Mean ± SEM).

Journal of Cell Science • Supplementary information

Table S1. Gene probes involved in cardiomyocyte differentiation are not significantly changed in SIRT1 KO E18.5 hearts

Click here to download Table S1

Table S2. Significantly changed Ingenuity Canonical Pathways in KO/WT E18.5 hearts

Click here to download Table S2

Journal of Cell Science • Supplementary information
