# Supplementary material for: SIRT1 regulates cardiomyocyte alignment during maturation
Source: J Cell Sci. 2022 Apr 1;135(7):jcs259076. doi: 10.1242/jcs.259076 (PMC9016619; doi:10.1242/jcs.259076)
Supplement: Supplementary file 1 [file joces-135-259076_review_history.pdf]

SIRT1 regulates cardiomyocyte alignment during maturation

Yi Fang, Wei Fan, Xiaojiang Xu, Agnes Janoshazi, David Fargo and Xiaoling Li
DOI: 10.1242/jcs.259076

Editor: Caroline Hill

Review timeline

Original submission:	29 June 2021
Editorial decision:	21 October 2021
First revision received:	24 January 2022
Accepted:	28 February 2022

Original submission

First decision letter

MS ID#: JOCES/2021/259076

MS TITLE: SIRT1 regulates cardiomyocyte alignment during maturation

AUTHORS: Yi Fang, Wei Fan, Xiaojiang Xu, Agnes Janoshazi, David Fargo, and Xiaoling Li
ARTICLE TYPE: Research Article

First of all I am so sorry that it has taken so long to get this paper reviewed. We had a lot of problems finding suitable reviewers. My sincere apologies.

We have now been able to reach a decision on the manuscript.

To see the reviewers' reports and a copy of this decision letter, please go to: https://submit-jcs.biologists.org and click on the 'Manuscripts with Decisions' queue in the Author Area. (Corresponding author only has access to reviews.)

As you will see, the reviewers think that your studies provide new insights into the regulation of cardiomyocyte maturation in the perinatal heart. However, they raise a number of substantial criticisms that prevent me from accepting the paper at this stage. They do suggest though that a revised version might prove acceptable, if you can address their concerns. In some cases, new experiments will be required. If you think that you can deal satisfactorily with the criticisms on revision, I would be pleased to see a revised manuscript. We would then return it to the reviewers.

We are aware that you may be experiencing disruption to the normal running of your lab that makes experimental revisions challenging. If it would be helpful, we encourage you to contact us to discuss your revision in greater detail. Please send us a point-by-point response indicating where you are able to address concerns raised (either experimentally or by changes to the text) and where you will not be able to do so within the normal timeframe of a revision. We will then provide further guidance. Please also note that we are happy to extend revision timeframes as necessary.

Please ensure that you clearly highlight all changes made in the revised manuscript. Please avoid using 'Tracked changes' in Word files as these are lost in PDF conversion.

I should be grateful if you would also provide a point-by-point response detailing how you have dealt with the points raised by the reviewers in the 'Response to Reviewers' box. Please attend to

© 2022. Published by The Company of Biologists under the terms of the Creative Commons Attribution License (https://creativecommons.org/licenses/by/4.0/).

all of the reviewers' comments. If you do not agree with any of their criticisms or suggestions please explain clearly why this is so.

Reviewer 1

Advance summary and potential significance to field

The report by Yi Fang et al., examines the effect of SIRT1 on cardiomyocyte alignment during maturation and development. The authors show a structural and cellular aberration of cardiac tissue of Sirt1 KO without a significant transcriptional effect, or prevention of differentiation into cardiomyocytes lineages. Rather changes appeared to be largely structural and related to myocardiac assembly as shown in Fig. 1 and Fig. 3, functionally causing irregular fibrillation of the assembled cardiac tissue. This process is rescued by genetical modification inducing Sirt1 expression in the Sirt1 KO after induction of cardiomyocyte differentiation, and is reported to be related to the deacetylase capacity of SIRT1 rather to the proteins presence in the cell.

Comments for the author

Major concerns:
1) The study supports that lack of Sirt1 activity is responsible for the impairments of cardiomyocyte maturation and alignment, which was confirmed by way of impacting several chemokine related pathways. However the molecular link was not directly established, as is even stated by the authors in the discussion. This lack of molecular data (i.e. ChIP of Sirt1 on one of the chemokines or assessment of possible deacetylated targets related to these chemokine axes), dampened my enthusiasm when reading the report. Could the authors provide some more direct SIRT1 related molecular confirmation to their structural observation?
2) Did authors observe a sex related effect in Figure 1 cardiac tissue for the KO? Have there been reports indicating such variations amongst sexes of Sirt1 nulls in regards to cardiac development?
3) Page 11 established that CXCR4 signaling is defective in the Sirt1 null mice due to CXCL12 production, which was not validated in-vivo- leaving this an unanswered question. Could this production be assessed in conditioned media by the cell lines? Or at least via immunoblot analysis of the myocardial extracts?
4) The connection between the CXCR4/ CXCL12 and CCR2/CCR4 is unclear (page 11 of the Results section), but it appears from the text that these two signaling pathways are temporally controlled during cardiomyocyte maturation. Could the authors add a schematic showing the temporal changes in the wt vs KO for these two chemotactic signaling pathways?
5) Page 12 jumps back to CXCR4/CXCL12 signaling but attempts to test CCL2 pathways- does the CCL2 pathway precede the CXCR4/CXCL12 pathways? This is conceptually unclear and could be defined in a scheme within Fig. 5, or Fig. 6.
6) The statistical evaluation of less than n=7 via t-test or Mann Whitney is inappropriate. Most sample sizes are between 3-4 and do not qualify for normality. If employing Mann Whitney test, it should precede with a Kruskal-Wallis test (equivalent to ANOVA in non parametric sample groups). Alternatively, a small sample group could bet tested for significance via the Wilcoxon U test, which does not require a Kruskal-
Wallis test before employing it in a paired analysis.

Minor Concerns:
1) Fig. 1C- could authors add an arrow to point on a Z-line within the micrograph or add an enlargement of a representative Z-line?
2) Graph bars as in 2E should display individual data points

Reviewer 2

Advance summary and potential significance to field

In this study, Fang et al investigated the role of SIRT1 in cardiomyocyte maturation. Using various in vitro and in vivo models, Fang et al showed that SIRT1 deficiency leads to impairment of cardiomyocyte/myofibril alignment, but not differentiation of cardiac progenitors. Further

© 2022. Published by The Company of Biologists under the terms of the Creative Commons Attribution License (https://creativecommons.org/licenses/by/4.0/).

mechanistic studies revealed that defective chemokine signaling, in particular the CXCL12/CXCR4 and CCL2/CCR2/CCR4 axis, may underlie the myofibril misalignment phenotype caused by SIRT1 loss-of-function. These studies provide new insights into the regulation of cardiomyocyte maturation in the perinatal heart.

Comments for the author

Major comments:
1. Assessment of myofibril/cardiomyocyte misalignment is key to this manuscript. However, detailed descriptions of these analyses are missing. How did the authors select the area of the heart for analysis (e.g., Figs 1A-C, 2H)? Were similar anatomical regions (e.g., LV vs RV, compact vs trabecular myocardium, base vs apex) of the heart analyzed in both WT and KO hearts? Similarly, in hESCs-CMs experiments, how are fields of interests selected? E.g., myofibril alignment differs greatly in KO D45 CMs between Figs 3D and E.
2. The use of a ubiquitous CreER (Figs 4C - H) does not support the claim that SIRT1 directly regulates cardiomyocyte remodeling in vivo (Page 10, lines 233 - 234). Along the same line, what are the cellular compositions in hESCs-CMs from d15 ? 30 of differentiation? It looks like that there are significant number of non-myocytes (ACTN2/GFP- cells in Fig 6C control). Could myofibril misalignment a consequence of SIRT1 deficiency in non-CMs?
A cardiomyocyte-specific Cre driver (with Sirt1floxed mice) would help answer this question.
3. Figs. 4H: Quantitative measurements of cardiomyocyte arrangement and/or myofibril misalignment are missing for this key piece of data.

Minor comments:
4. Fig. 2C: some of the asterisks are misplaced.
5. Fig. 2E: Are there significant difference in the number of Nkx2.5-eGFP+ cells between WT and KO iCMs/well assayed by FACS? Quantification of the number of CMs would be a better readout than qPCR of sorted eGFP+ cells for cardiomyocyte differentiation from progenitors.
6. Fig. 3B: It would be useful to explain how each category is defined.
7. Figs 4A ? B: A GFP transfection (or equivalent) control is needed. Despite the statistical difference between WT and HY (Fig 4B), both forms of SIRT1 were able to significantly rescue the beating frequency phenotype compared with SIRT1 KO cells (compare Figs 4B and 3H KO). These data argue against the importance of the deacetylase activity of SIRT1 in normal remodeling of iCMs (Page 9, line 211).
8. Fig 5D: Similar to comment#1, how are the field of interests selected? It looks like the sarcomeric staining was only located in the compact layer myocardium (Flox), which is not present in the representative image of TiKO?
9. Authors should consider using dot plots instead bar graphs, which better present the distribution of data points.

First revision

<u>Author response to reviewers' comments</u>

Reviewer 1 Advance summary and potential significance to field
The report by Yi Fang et al., examines the effect of SIRT1 on cardiomyocyte alignment during maturation and development. The authors show a structural and cellular aberration of cardiac tissue of Sirt1 KO without a significant transcriptional effect, or prevention of differentiation into cardiomyocytes lineages. Rather changes appeared to be largely structural and related to myocardiac assembly as shown in Fig. 1 and Fig. 3, functionally causing irregular fibrillation of the assembled cardiac tissue. This process is rescued by genetical modification inducing Sirt1 expression in the Sirt1 KO after induction of cardiomyocyte differentiation, and is reported to be related to the deacetylase capacity of SIRT1 rather to the proteins presence in the cell.

© 2022. Published by The Company of Biologists under the terms of the Creative Commons Attribution License (https://creativecommons.org/licenses/by/4.0/).

Reviewer 1 Comments for the author

Major concerns:

1)The study supports that lack of Sirt1 activity is responsible for the impairments of cardiomyocyte maturation and alignment, which was confirmed by way of impacting several chemokine related pathways. However, the molecular link was not directly established, as is even stated by the authors in the discussion. This lack of molecular data (i.e. ChIP of Sirt1 on one of the chemokines or assessment of possible deacetylated targets related to these chemokine axes), dampened my enthusiasm when reading the report. Could the authors provide some more direct SIRT1 related molecular confirmation to their structural observation?

Thanks for the suggestion. We have been working very hard to establish possible molecular mechanism(s) underlying SIRT1-mediated transcriptional regulation of chemokine gene expression as the reviewer suggested during the revision. We now showed that SIRT1 is enriched on the promoter of CCL2 gene in WT iCMs, and the peak locus is overlapped with a locus also peaked with H3K27ac, a histone activation mark. However, this enrichment was blunted in KO iCMs (new Figure 6D). We further showed that inhibition of HDACs by TSA significantly rescued the defective expression of CCL2, CCR2, and CCR4 genes in the KO iCMs (new Figure 6E).

We tried several candidate transcription factors (potential deacetylation substrates of SIRT1) that are reported or predicted to bind near the SIRT1 peak locus on the CCL2 promoter in iCMs, but none of them showed significant acetylation in these cells. Nevertheless, given the reports that SIRT1 or deacetylation represses HDAC1 activity and thus promoting gene expression (Binda et al., 2008; Qiu et al., 2006), our current observations suggest a model in which SIRT1 promotes an open configuration of chromatin on the promoters of genes in the CCL2 signaling pathway via inhibiting HDACs, which in turn allows histone acetylation and transcriptional activation of these genes. We added a sentence in Discussion (in Line 396-402) to clarify this point: “Intriguingly, our data indicate that SIRT1 positively modulates the chemokine signaling during cardiomyocyte remodeling and promotes cardiomyocyte alignment. Specifically, we show that SIRT1 enhances the promoter histone acetylation levels and activates the expression of CCL2 by repressing HDACs (Fig. 6D-6E), which are in line with previous reports that SIRT1 or deacetylation repress HDAC1 activity (Binda et al., 2008; Qiu et al., 2006).”

I hope our new data and discussion addressed, at least partially, the reviewer’s concern.

2)Did authors observe a sex related effect in Figure 1 cardiac tissue for the KO? Have there been reports indicating such variations amongst sexes of Sirt1 nulls in regards to cardiac development?

No, we did not observe any sex-related effect in cardiac tissue for SIRT1 KO embryos. We collected the WT and KO E18.5 hearts from both genders for the same morphological analysis. We also didn’t see any reports indicating sex related effects of Sirt1 regarding cardiac development.

We added two sentences on Materials and Methods, Animal Studies, to indicate that “Hearts from both sexes were analyzed.”.

3)Page 11 established that CXCR4 signaling is defective in the Sirt1 null mice due to CXCL12 production, which was not validated in-vivo- leaving this an unanswered question. Could this production be assessed in conditioned media by the cell lines? Or at least via immunoblot analysis of the myocardial extracts?

We apologized for the lack of in vivo validation for CXCL12/CXCR4. We did an immunofluorescence staining for CXCL12 in WT and Sirt1 KO hearts for the revision, which indicated that the protein levels of CXCL12 was lower in SIRT1 deficient hearts in vivo. This piece of new result is now included in new Figure 5D, left panels.

4)The connection between the CXCR4/ CXCL12 and CCR2/CCR4 is unclear (page 11 of the Results section), but it appears from the text that these two signaling pathways are temporally controlled during cardiomyocyte maturation. Could the authors add a schematic showing the temporal changes in the wt vs KO for these two chemotactic signaling pathways?

© 2022. Published by The Company of Biologists under the terms of the Creative Commons Attribution License (https://creativecommons.org/licenses/by/4.0/).

Yes, we added a cartoon model in new Figure 6F to show the temporal expression of the two signaling pathways during in vitro cardiomyocyte differentiation from hESCs.

5)Page 12 jumps back to CXCR4/CXCL12 signaling but attempts to test CCL2 pathways- does the CCL2 pathway precede the CXCR4/CXCL12 pathways? This is conceptually unclear and could be defined in a scheme within Fig. 5, or Fig. 6.

From the expression data in Figure 5A, we didn't see that the CCL2 pathway precedes the CXCR4/CXCL12 pathway. Instead, it appears that during cardiomyocyte differentiation, the CXCR4/CXCL12 pathway was induced earlier than the CCL2 pathways (although CXCL12 was reduced on d8), whereas the CCL2 pathways are more dramatically induced than the CXCR4/CXCL12 pathway. Again, as suggested by the reviewer, we added a cartoon model in new Figure 6F to show the temporal expression of the two signaling pathways during in vitro cardiomyocyte differentiation from hESCs.

6)The statistical evaluation of less than n=7 via t-test or Mann Whitney is inappropriate. Most sample sizes are between 3-4 and do not qualify for normality. If employing Mann Whitney test, it should precede with a Kruskal-Wallis test (equivalent to ANOVA in non parametric sample groups). Alternatively, a small sample group could best tested for significance via the Wilcoxon U test, which does not require a Kruskal-Wallis test before employing it in a paired analysis.

Many thanks for the suggestion on the statistical analysis of our data. However, several points that the reviewer raised here are confusing: (1) The rationale of performing paired analysis on data that were not designed to be matched is not clear. (2) Per a professional statistician, Mann Whitney U test is the same as Wilcoxon rank-sum test, which does not require a Kruskal-Wallis test. (3) The assumption of the t-test is that the underlying population has a normal distribution. We believe that most of the measurements we considered in this paper are known to follow a normal distribution (continuous not binary) and the t-test is a valid test. Unfortunately with a sample size of 3 or 4 we do not have enough power to perform non-parametric test.

To address the reviewer concern, we listed the small sample size as a limitation of our study on Page 16 (Line 410-412). In addition, we redid our statistical analysis on data with more than two comparison groups using 2-way ANOVA with adjustments on multiple comparisons.

We hope our response here addressed the reviewer's concern.

Minor Concerns:
1)Fig. 1C- could authors add an arrow to point on a Z-line within the micrograph or add an enlargement of a representative Z-line?

Thanks for the suggestion. We added arrowheads to point on Z-lines within the micrograph.

2)Graph bars as in 2E should display individual data points.

Thanks for the suggestion, we remade all but one of our bar figures as dot plots so that individual data points will be displayed. The only exception is Figure 3H. The data was independently analyzed by a core-facility specialist who left the institute last year. We were not able to locate her original data sheet. We apologize for this situation.

Reviewer 2 Advance summary and potential significance to field
In this study, Fang et al investigated the role of SIRT1 in cardiomyocyte maturation. Using various in vitro and in vivo models, Fang et al showed that SIRT1 deficiency leads to impairment of cardiomyocyte/myofibril alignment, but not differentiation of cardiac progenitors. Further mechanistic studies revealed that defective chemokine signaling, in particular the CXCL12/CXCR4 and CCL2/CCR2/CCR4 axis, may underlie the myofibril misalignment phenotype caused by SIRT1 loss-of-function. These studies provide new insights into the regulation of cardiomyocyte maturation in the perinatal heart.

© 2022. Published by The Company of Biologists under the terms of the Creative Commons Attribution License (https://creativecommons.org/licenses/by/4.0/).

Reviewer 2 Comments for the author

Major comments:

1. Assessment of myofibril/cardiomyocyte misalignment is key to this manuscript. However, detailed descriptions of these analyses are missing. How did the authors select the area of the heart for analysis (e.g., Figs 1A-C, 2H)? Were similar anatomical regions (e.g., LV vs RV, compact vs trabecular myocardium, base vs apex) of the heart analyzed in both WT and KO hearts? Similarly, in hESCs-CMs experiments, how are fields of interests selected? E.g., myofibril alignment differs greatly in KO D45 CMs between Figs 3D and E.

We apologize that we did not provide detailed descriptions on our myofibril/cardiomyocyte misalignment assay. Yes, for myofibril/cardiomyocyte misalignment analysis in embryonic mouse hearts, we selected comparable areas from the same anatomical regions (LV, compact myocardium) in both WT and KO hearts. In Figure 1A and B, cross sections towards to the base of LV were shown, whereas in Figure 4H, the longitudinal sections localized to the middle of LV were shown. We added the information in the figure legends.

In hESCs-CMs experiments, cardiomyocytes first appeared as clumps, and then well-aligned cardiac fibers were formed between clumps or around clumps. We chose the fiber fields between clumps for the alignment analysis (as elucidated in cartoon of new Figure 3B). Both Figure 3D and 3E are from the fiber regions (as indicated by the red box in cartoon of new Figure 3B), except that Figure 3D was taken at a higher magnification whereas Figure 3E displayed a bigger field. Importantly, in both Figure 3D and 3E, SIRT1 KO cardiomyocytes lack a clear alignment along one axis.

2. The use of a ubiquitous CreER (Figs 4C - H) does not support the claim that SIRT1 directly regulates cardiomyocyte remodeling in vivo (Page 10, lines 233 - 234). Along the same line, what are the cellular compositions in hESCs-CMs from d15 ? 30 of differentiation? It looks like that there are significant number of non-myocytes (ACTN2/GFP- cells in Fig 6C control). Could myofibril misalignment a consequence of SIRT1 deficiency in non-CMs?
A cardiomyocyte-specific Cre driver (with Sirt1floxed mice) would help answer this question.

We agree with the reviewer that a cardiomyocyte-specific Cre driver would be a better model to determine cardiomyocyte-specific functions of Sirt1 in cardiac development. However, the most extensively utilized tamoxifen-inducible cardiac-specific cre line, αMHC-cre-CreERT2 (also called αMHC-MerCreMer), is not suitable for our study, as αMHC is the main MHC isoform in the adult murine ventricle (Sohal et al., 2001). Its expression is very low in fetal ventricle, and during embryonic development, it only labels cardiomyocytes of low-cycling capacity. Also, although there are a few additional inducible cardiomyocyte-specific Cre lines (e.g. Tnnt2-rtTA/tetO mice) that are available for us to try, it will take many months to even breed them with Sirt1 floxed mice to generate inducible cardiomyocyte-specific Sirt1 KO lines, which is beyond the normal revision time frame.

For the in vitro models, yes, there are non-cardiomyocytes present in the hESCs-CMs. So it is possible that myofibril misalignment a consequence of SIRT1 deficiency in non-CMs.

To address the reviewer’s concern, we revised our conclusion on Page 10 into the following: “Therefore, SIRT1 regulates cardiomyocyte remodeling at late developmental stages both in vitro and in vivo.” We also listed lacking cardiomyocyte-specific SIRT1 KO model as a limitation of our study in the Discussion (Line 403-410): “Our study has some limitations. Firstly, we noticed that SIRT1 and the induced chemokines are expressed in cardiomyocytes as well as many other cell types in both mouse embryonic hearts and the in vitro cardiomyocyte differentiation system. Therefore, the universal manipulation of SIRT1/CCL2 signaling in our study did not allow us to dissect the cell type-specific functions of these genes during cardiomyocyte remodeling. Future studies with cell-type specific gene manipulations (e.g. cardiomyocyte-specific deletion of SIRT1) are required to further determine their specific role in cardiomyocytes.”

3. Figs. 4H: Quantitative measurements of cardiomyocyte arrangement and/or myofibril misalignment are missing for this key piece of data.

© 2022. Published by The Company of Biologists under the terms of the Creative Commons Attribution License (https://creativecommons.org/licenses/by/4.0/).

Thanks for the suggestion, we did the quantification analysis as suggested, and the results are now shown at the right of Figure 4H.

Minor comments:
4. Fig. 2C: some of the asterisks are misplaced.

Thanks. We corrected the placing of the asterisks.

5. Fig. 2E: Are there significant difference in the number of Nkx2.5-eGFP+ cells between WT and KO iCMs/well assayed by FACS? Quantification of the number of CMs would be a better readout than qPCR of sorted eGFP+ cells for cardiomyocyte differentiation from progenitors.

The purpose of Figure 2E was to determine the possible differential expression of candidate genes in WT and SIRT1 KO cardiomyocytes without the impact from differentiation efficiency.

In terms of differentiation efficiency, we did observe some delay of Nkx2.5-eGFP+ cells from SIRT1 KO hESCs before d6 by FACS and qPCR (data not shown). From our qPCR analysis of cardiomyocyte-specific markers from total cells in Figure 2C, we could still see the differential expression of some genes between WT and SIRT1 KO cells on d8. However, by d20, the expression of most of the genes was comparable between WT and KO. In particular, the expression of NKX2.5, which drives eGFP reporter expression, was comparable between WT and SIRT1 KO cells.

6. Fig. 3B: It would be useful to explain how each category is defined.

Thanks for the suggestion, the definition of each category was explained in the legend. We added a cartoon in Figure 3B to illustrate it for the revision.

7. Figs 4A ? B: A GFP transfection (or equivalent) control is needed. Despite the statistical difference between WT and HY (Fig 4B), both forms of SIRT1 were able to significantly rescue the beating frequency phenotype compared with SIRT1 KO cells (compare Figs 4B and 3H KO). These data argue against the importance of the deacetylase activity of SIRT1 in normal remodeling of iCMs (Page 9, line 211).

Our cardiomyocytes are already labeled with eGFP under the promoter of NKX2.5. Did the reviewer suggest doing an empty vector control? Thanks, we did this control and the new results were added into new Figure 4A-B.

Yes, the reviewer is correct that SIRT1 HY mutant can also partially rescue the beating frequency phenotype of KO cardiomyocytes. Our empty vector control indicates that AAV viral infection alone have some mild affects on the remodeling of iCMs, so the impact of HY mutant on the remodeling phenotype could come from the viral infection.

8. Fig 5D: Similar to comment#1, how are the field of interests selected? It looks like the sarcomeric staining was only located in the compact layer myocardium (Flox), which is not present in the representative image of TiKO?

As explained above, we chose similar anatomical regions of the heart between WT and KO hearts. All hearts in Figure 5D were longitudinally sectioned. However, due to the distinct orientations of different myofibrils, both cross sections and longitudinal sections of myofibrils were observed in the same image. In the figure, we primarily compared the Ccl12 staining in the trabecular regions from both WT and KO hearts, which showed that in the WT hearts, Ccl12 signals were present in all the myocytes throughout the sections. However, in the KO hearts, Ccl12 signals were much lower in the center of tissues, although remain strong in the endocardial cells.

In the original figure, we also enlarged an area in the longitudinal section in a WT heart to show an example of localization of Ccl12 in sarcomeric structures in some cells. We apologize that this caused confusion. We deleted the enlarged images for the revision.

9. Authors should consider using dot plots instead bar graphs, which better present the distribution of data points.

© 2022. Published by The Company of Biologists under the terms of the Creative Commons Attribution License (https://creativecommons.org/licenses/by/4.0/).

Thanks for the suggestion, we remade all but one of our bar figures as dot plots so that individual data points will be displayed. The only exception is Figure 3H. The data was independently analyzed by a core-facility specialist who left the institute last year. We were not able to locate her original data sheet. We apologize for this situation.

References:

Binda, O., Nassif, C., and Branton, P.E. (2008). SIRT1 negatively regulates HDAC1-dependent transcriptional repression by the RBP1 family of proteins. Oncogene 27, 3384-3392. 10.1038/sj.onc.1211014.
Qiu, Y., Zhao, Y., Becker, M., John, S., Parekh, B.S., Huang, S., Hendarwanto, A., Martinez, E.D., Chen, Y., Lu, H., et al. (2006). HDAC1 acetylation is linked to progressive modulation of steroid receptor-induced gene transcription. Mol Cell 22, 669-679. 10.1016/j.molcel.2006.04.019.
Sohal, D.S., Nghiem, M., Crackower, M.A., Witt, S.A., Kimball, T.R., Tymitz, K.M., Penninger, J.M., and Molkentin, J.D. (2001). Temporally regulated and tissue-specific gene manipulations in the adult and embryonic heart using a tamoxifen-inducible Cre protein. Circ Res 89, 20-25. 10.1161/hh1301.092687.

Second decision letter

MS ID#: JOCES/2021/259076

MS TITLE: SIRT1 regulates cardiomyocyte alignment during maturation

AUTHORS: Yi Fang, Wei Fan, Xiaojiang Xu, Agnes Janoshazi, David Fargo, and Xiaoling Li
ARTICLE TYPE: Research Article

I am happy to tell you that your manuscript has been accepted for publication in Journal of Cell Science, pending standard ethics checks.

Reviewer 1

Advance summary and potential significance to field

The Authors addressed my concerns sufficiently

Comments for the author

The Authors addressed my concerns sufficiently

Reviewer 2

Advance summary and potential significance to field

This revised version has provided clearer explanation of experimental methods and potential molecular mechanisms downstream of SIRT1 in regulating cardiomyocyte alignment during maturation.

Comments for the author

The authors have responded to my previous comments/suggestions satisfactorily. I have no further questions/comments. Congratulations on the nice work!

© 2022. Published by The Company of Biologists under the terms of the Creative Commons Attribution License (https://creativecommons.org/licenses/by/4.0/).
